# Natural compounds target the M23B zinc metallopeptidase Mpg to modulate *Neisseria gonorrhoeae* Type IV pilus expression

**DOI:** 10.1128/mbio.04027-24

**Published:** 2025-02-25

**Authors:** Kathleen R. Nicholson, Shaohui Yin, Jennifer L. Edwards, Chi-Hao Luan, H Steven Seifert

**Affiliations:** 1 Department of Microbiology-Immunology, Feinberg School of Medicine, Northwestern University, Chicago, Illinois, USA; 2 Center for Microbial Pathogenesis, Abigail Wexner Research Institute at Nationwide Children’s Hospital, The Ohio State University, Columbus, Ohio, USA; 3 High Throughput Analysis Laboratory and Department of Molecular Biosciences, Northwestern University, Evanston, USA; The Ohio State University College of Nursing, Columbus, Ohio, USA

**Keywords:** *Neisseria gonorrhoeae*, pilus, metallopeptidase, small molecule screen, natural compounds

## Abstract

*Neisseria gonorrhoeae* uses the Type IV pilus (T4p) to colonize several sites within humans by adhering to host cells and tissues. Previously, we identified a periplasmic M23B zinc metallopeptidase, Mpg, that is necessary to protect from oxidative and nonoxidative killing and these phenotypes are mediated by Mpg activities on T4p expression. Here, we use a high-throughput, target-based screening approach to identify novel inhibitors of Mpg’s enzymatic activity. We identified two natural compounds, punicalagin and chebulinic acid, which inhibit the peptidoglycan-hydrolyzing activity of Mpg in a dose-dependent manner. Moreover, treatment of *N. gonorrhoeae* with these compounds leads to a concomitant decrease in the number of T4p, similar to an *mpg* mutant. However, these compounds are not toxic to *N. gonorrhoeae*. These compounds exhibit activity against Mpg orthologs from other bacterial species. Notably, these natural compounds inhibit *N. gonorrhoeae* colonization and survival in cell culture models of infection. This work provides the characterization of two natural compounds with activity against *N. gonorrhoeae* T4p through the Mpg M23B class zinc metallopeptidase.

**IMPORTANCE:**

*Neisseria gonorrhoeae* is a global health burden with high transmission rates and multidrug resistance. *N. gonorrhoeae* encodes a Type IV pilus (T4p), which is a major colonization and virulence factor. The importance of the T4p in multiple stages of infection makes it an attractive drug target. Previously, we identified an M23B zinc metallopeptidase, Mpg, important for T4p production and T4p-mediated resistance to neutrophil killing. In this study, we identified two natural compounds, punicalagin and chebulinic acid, as novel inhibitors of Mpg’s enzymatic activity that thus inhibit T4p expression. These findings identify two potential anti-colonization and anti-virulence compounds and provide a framework to target T4p components for future screens, poising the field to potentially discover additional compounds to combat *N. gonorrhoeae* infection.

## INTRODUCTION

*Neisseria gonorrhoeae* is the most common causative agent of gonorrhea in humans, while a specific clade of *Neisseria meningitidis* is also a rising cause of urethritis that is indistinguishable from gonococcal gonorrhea (1, 2). Most gonorrhea infections are asymptomatic, leading to high transmission rates (1). However, long-term gonorrhea infections in women lead to severe reproductive health outcomes, including pelvic inflammatory disease, infertility, ectopic pregnancies, and first-trimester abortion (3, 4). Untreated infections in women or men can result in disseminated gonococcal infection, which has serious complications like infectious arthritis and endocarditis (5). Rates of drug resistance in *N. gonorrhoeae* continue to rise, with widespread resistance emerging to the current first-line recommended antibiotic, ceftriaxone (6–9). Moreover, previous infection with *N. gonorrhoeae* does not lead to immunity to future infections (10).

Gonococcal infection is defined by three steps: adherence, colonization, and invasion (11). The first step of colonization, adherence to the mucosal epithelium, requires the Type IV pilus (T4p) (12). The T4p is a critical *N. gonorrhoeae* virulence factor that mediates DNA uptake, enables twitching motility, promotes adhesion to host cells, and prevents polymorphonuclear cell (PMN) killing (13–16). *N. gonorrhoeae* recovered from infected patients are piliated, even though piliation is lost during *in vitro* cultivation, illustrating the importance of this virulence factor (17–20). After initial adherence, the T4p continues to mediate pathogenesis through resistance to host neutrophil killing (15). Given its role in two major steps of pathogenesis, the T4p is an attractive target for novel antimicrobial compounds to treat gonorrhea.

Two previous studies have aimed at discovering new antimicrobial compounds targeting the T4p of *Neisseria meningitidis* (21, 22). One of these studies identified trifluoperazine and thioridazine, which belong to the phenothiazine family (22). Treatment with trifluoperazine and thioridazine reduced T4p function *in vitro* and *in vivo* and led to reduced pathology (22). These compounds did not directly target the T4p but instead impacted the NADH-ubiquinone oxidoreductase complex, which led to downstream piliation defects (22). Another study identified three compounds with activity against the T4p PilF extension ATPase (called PilB in most other organisms), preventing the assembly of the T4p (21).

We previously identified an M23B class zinc metallopeptidase, Mpg (product of the *NGO_1686* locus), that is required for full piliation (15, 16). M23B zinc metallopeptidases are located in the periplasm and are involved in a variety of bacterial processes impacting peptidoglycan (PG) modifications, including PG crosslinking and cleavage of septal PG (23–28). Mpg exhibits a zinc-dependent carboxypeptidase and an endopeptidase activity, which do not impact cell morphology but impact pilus biogenesis (16). Mpg is an important factor for the pilus-mediated resistance to PMN killing through its role in pilus biogenesis (15). Mpg may be an attractive drug target since inhibiting Mpg would affect both colonization and sensitivity to PMN killing. Here, we used high-throughput target-based screening to target the T4p by identifying Mpg inhibitors.

## RESULTS

### A screen for compounds with activity against Mpg

The fluorescence thermal shift (FTS) assay is a versatile *in vitro* binding assay, useful for identifying protein-ligand interactions in both primary high-throughput screening (HTS) and secondary assays to confirm hit compounds from functional assays (29, 30). For Mpg, although a functional assay exists, the slow and complex nature of the PG digestion process makes it impractical for large-scale compound screening. To address this issue, we developed an FTS assay suitable for HTS applications with Mpg.

FTS monitors protein thermal denaturation using an environment-sensitive dye, such as Sypro-Orange, which fluoresces upon binding to hydrophobic regions exposed during protein unfolding. Small molecule binding can stabilize or destabilize the target protein, causing a measurable shift in its thermal denaturation (melting) temperature (T_m_). The recombinant Mpg protein exhibits a suitable unfolding profile for FTS-based primary screening in HTS. Sypro-Orange is excited at 473 nm and emits fluorescence at 610 nm when it binds to protein hydrophobic regions typically buried in the native protein structure. Comparison of the thermal denaturation profiles of Mpg in the presence and absence of hit compounds revealed destabilization of the native protein structure, confirming compound binding but not the inhibition of enzymatic activity.

### Results of the small molecule screen for Mpg inhibitory compounds

Using the FTS-based assay, we screened a total of 37,760 compounds in the Northwestern High Throughput Analysis Laboratory’s compound library: 1,280 molecules from the protein-protein interaction inhibitors collection, 11,520 from the kinase inhibitors collection, 20,480 from the diverse chemical structures collection, 2,880 bioactive compounds, and 1,600 compounds from the cell cycle/DNA damage collection. In total, 182 compounds were classified as primary hits, defined by an absolute thermal shift (|ΔT_m_|) greater than 1.0°C, resulting in a hit rate of 0.48%.

**Fig 1:**
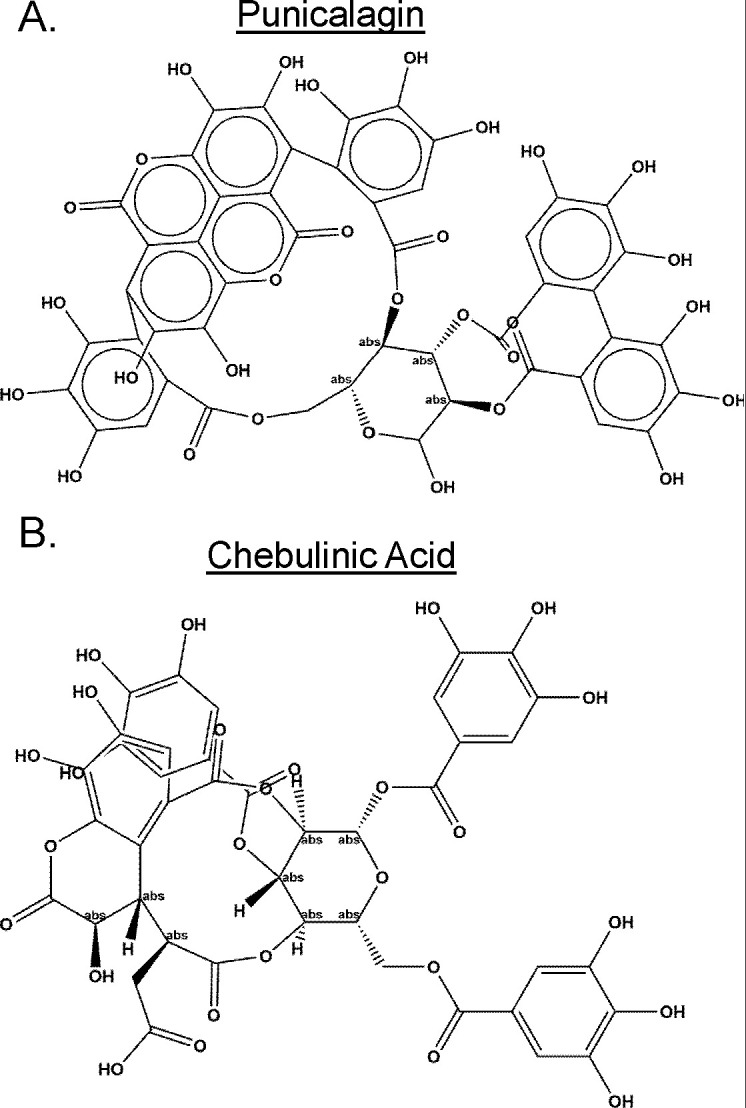
Punicalagin and chebulinic acid chemical structures. (**A)** Chemical structure of punicalagin and (**B)** chebulinic acid.

**Fig 2:**
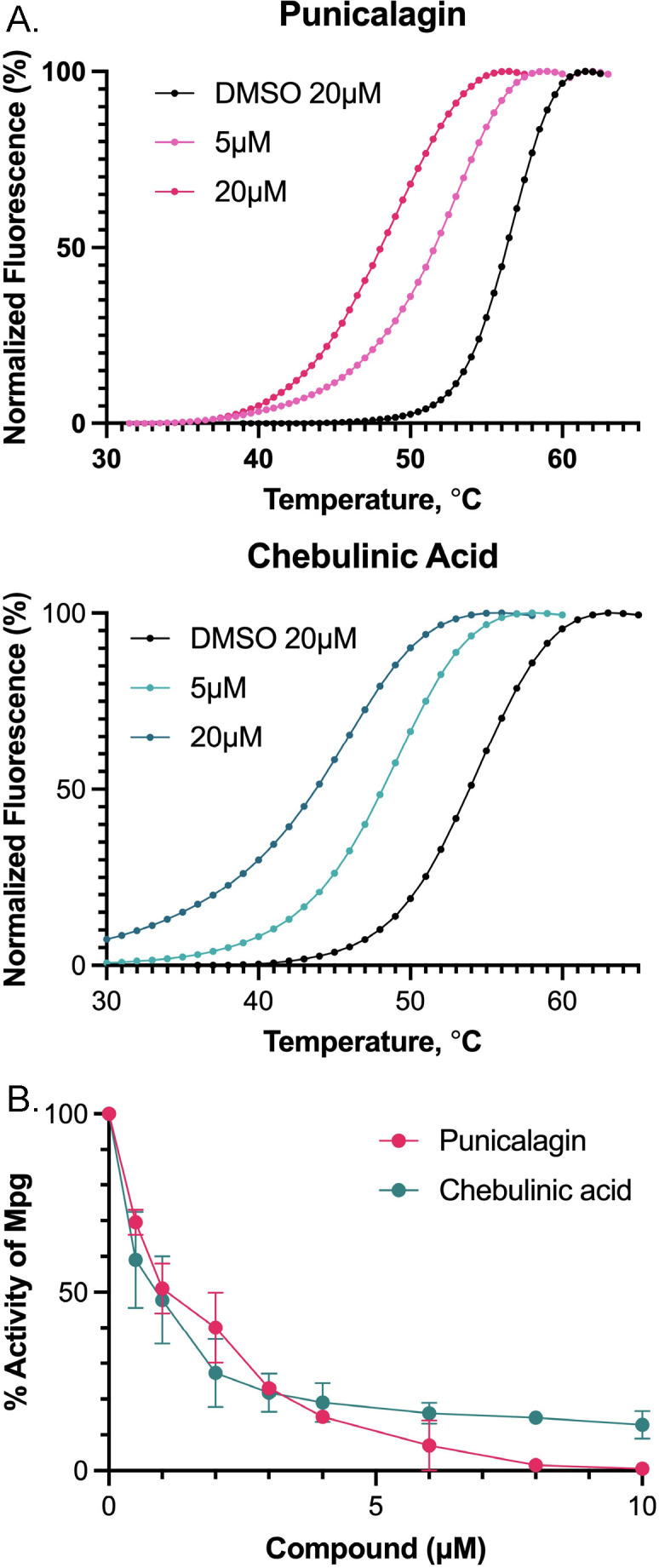
Sypro-Orange detection of punicalagin and chebulinic acid Mpg melting curves. (**A)** Normalized compound melting curves with the dimethyl sulfoxide (DMSO) control. (**B)** PG dye release assay with an Mpg dose response. 0.5–10 µM of punicalagin or chebulinic acid reacted with 1 µM Mpg.

The top primary hits were further evaluated in a compound dose-response assay using FTS to measure T_m_ shifts. We identified punicalagin and chebulinic acid as two compounds of interest (Fig. 1A and B). Punicalagin and chebulinic acid are naturally occurring compounds belonging to the ellagitannin family. Punicalagin is abundant in pomegranates, while chebulinic acid is found in fruit from the *Terminalia chebula* tree (31, 32). Punicalagin and chebulinic acid exhibited dose-dependent Tm shifts (Fig. 2A). Punicalagin caused a −4.4°C shift, while chebulinic acid resulted in a −2.9°C shift, both at 5 µM concentration. The negative Tm shifts suggest that the binding of these hit compounds destabilizes Mpg.

To determine if the interaction of punicalagin or chebulinic acid with Mpg resulted in a loss of Mpg function, we performed a secondary *in vitro* enzymatic assay. We used dye-labeled PG to monitor the release of soluble dye-labeled PG from the polymer as a measure of the PG-hydrolyzing activity of Mpg and the effect of punicalagin or chebulinic acid on Mpg activity. There was a dose-dependent decrease in the *in vitro* PG-hydrolyzing activity of 1 µM Mpg when treated with up to 10 µM punicalagin or chebulinic acid (Fig. 2B). One micromolar of either compound produced an approximately 50% reduction in Mpg activity with most of the activity abolished at 10 µM of the compounds (Fig. 2B).

We screened several related natural compounds for activity against Mpg (Fig. S1A). Punicalagin and chebulinic acid showed the most significant decrease in Mpg activity, while geranin and tannic acid showed moderate efficacy against Mpg (Fig. S1A and B). Additionally, we tested punicalagin derivatives for activity against Mpg (Fig. S2A) (33). These derivatives, except ellagic acid, did not show activity against Mpg when tested in isolation; however, when tested in pairs, some derivatives exhibited a 50% reduction in Mpg activity (Fig. S2B). These results show that punicalagin and chebulinic acid are potent inhibitors of Mpg activity.

### Natural compounds punicalagin and chebulinic acid bind to Mpg

The FTS assay does not provide information about specific binding sites or confirm whether the compounds directly bind to Mpg. To better understand the mode of interaction between the hit compounds and Mpg, we performed molecular docking using AutoDock4 (34) and AutoDock Vina (35) with the crystal structure of Mpg (PDB 6muk) (36). In a blind search, each hit compound was positioned across the surface of Mpg in a grid scan to identify potential binding sites. As shown in Fig. S3A, three binding sites were identified. Site 1 is located at the Zn²^+^-containing active site, conserved in the peptidase M23B family (37). The other two sites are positioned on the backside of Site 1, within a long groove. Site 1 residues are part of a conserved motif that coordinates the Zn²^+^ ion (Fig. S3A; Table S1). Our working model suggests that this Zn²^+^ active site connects to a larger binding area capable of accommodating the backbone N-acetylglucosamine and N-acetylmuramic acid disaccharide, the repeating unit in PG (Table S1). This area features a deep cleft distal to the Zn²^+^ ion. Site 2 is adjacent to Site 1, while Site 3 lies at the end of the long groove (Fig. S3A; Table S1). Therefore, molecules binding in this region may interfere with the interaction between Mpg and PG, potentially impacting endopeptidase activity. Although binding at Sites 2 and 3 may not directly block the Zn²^+^ active site, they could hinder Mpg’s enzymatic activity by impeding PG binding (Fig. S3A).

In addition, we used AlphaFold to predict the interaction between Mpg and punicalagin or chebulinic acid (Fig. S3B) (38). To avoid bias, we only provided the Mpg sequence and compound structure without specifying the protein identity or active site location. The predicted binding results corroborated our findings from the AutoDock/Vina grid scans, further validating the identified interaction sites.

### Punicalagin and chebulinic acid have activity against Mpg orthologs

**Fig 3:**
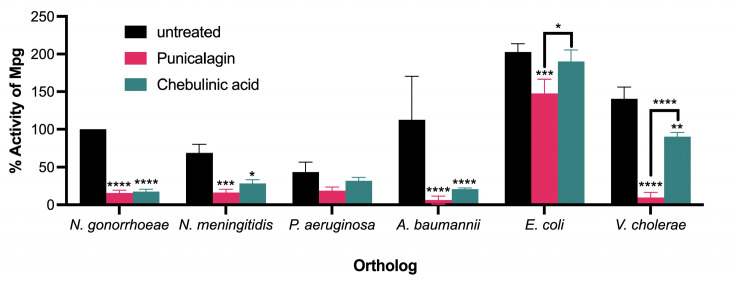
Punicalagin and chebulinic acid show inhibitory activity against Mpg orthologs. Mpg orthologs from gram-negative species: *N. gonorrhoeae* FA1090, *N. meningitidis* MC58, *P. aeruginosa* PAO1, *A. baumannii* Ab2, *E. coli* K12 MG1655, and *V. cholerae* O395 VC1. Each reaction contained 1 µM Mpg, 10 µM punicalagin or Chebulinic acid, and 10 mg/mL Remazol brilliant blue (RBB)-labeled gonococcal PG. Percent activity is represented relative to untreated Mpg activity. Statistical analysis was performed using a two-way analysis of variance (ANOVA) with multiple comparisons. Significance is shown relative to untreated Mpg from each bacterial ortholog. *N. gonorrhoeae*: ****P < 0.0001. *N. meningitidis*: ***P = 0.0004; *P = 0.0397. *A. baumannii*: ****P < 0.0001. *E. coli*: ***P = 0.0003. *V. cholerae*: ****P < 0.0001; **P = 0.0076. Bracketed significance shows comparisons between treatments within brackets. *E. coli* punicalagin vs. chebulinic acid: *P = 0.0413. *V. cholerae* punicalagin vs. chebulinic acid: ****P < 0.0001.

M23B class zinc metallopeptidases are found in most bacterial species. We wanted to determine whether compounds active against the *N. gonorrhoeae* Mpg are also active against Mpg orthologs. We chose Mpg orthologs in five gram negative bacteria encoding T4p for further testing, including *Pseudomonas aeruginosa*, *Acinetobacter baumannii*, *Escherichia coli*, and *Vibrio cholerae*. These sequences exhibited conservation in the C-terminal LytM domain, which contains the active site required for PG hydrolysis (Fig. S4) (15). Orthologous Mpg proteins were expressed and purified for *in vitro* enzymatic assays. Each purified M23B ortholog was assayed for PG hydrolysis using the dye release assay, where untreated orthologs served as internal positive controls for activity (Fig. 3). *N. gonorrhoeae* Mpg served as a positive control for punicalagin and chebulinic acid treatment (Fig. 3). The *N. meningitidis* and *A. baumannii* orthologs showed significant reductions in PG hydrolysis upon treatment with punicalagin or chebulinic acid. The *P. aeruginosa* ortholog did not show significant reductions in PG hydrolysis activity, though activity was reduced upon treatment with punicalagin and chebulinic acid. Interestingly, the *E. coli* and *V. cholerae* orthologs were more sensitive to punicalagin than chebulinic acid.

In gram-positive bacteria, we tested two *Staphylococcus aureus* Mpg orthologs, LytM1 and a glycine-glycine (G-G) endopeptidase, using dye-labeled *S. aureus* PG as the substrate (Fig. S5; Fig. S6). Untreated LytM1 and the G-G endopeptidase were internal controls for PG hydrolytic activity. Punicalagin and chebulinic acid showed less significant activity against the LytM1 protein and more significant activity against the G-G endopeptidase (Fig. S6). The inhibitory activity of the compounds against different Mpg orthologs suggests that these natural compounds may show activity against other bacterial species.

### Treatment with punicalagin or chebulinic acid reduces *N. gonorrhoeae* piliation

**Fig 4:**
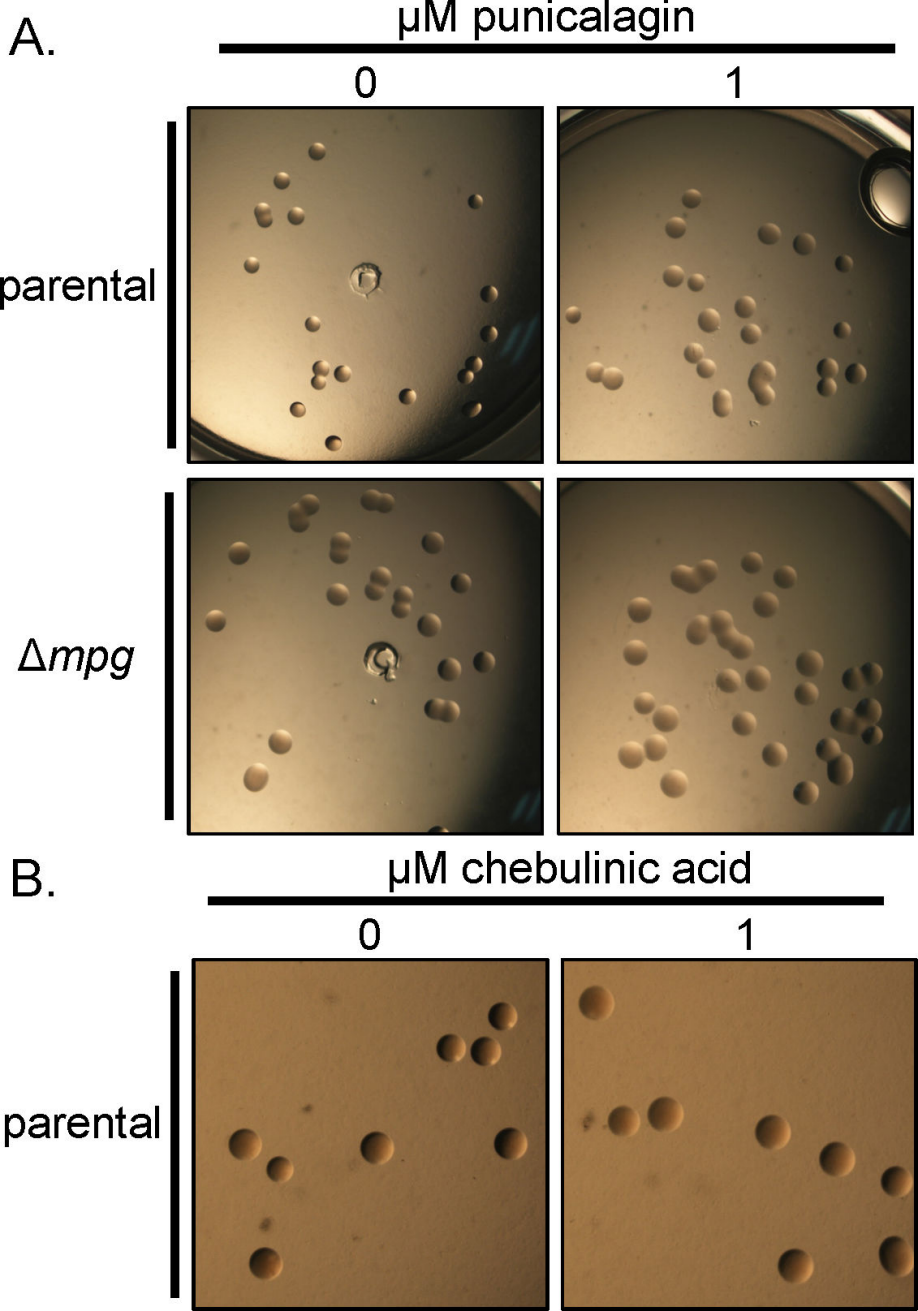
Treatment of *N. gonorrhoeae* with punicalagin or chebulinic acid reduces piliation colony morphologies. Colony morphology of *N. gonorrhoeae* grown with 1 µM of (A) punicalagin or (**B)** chebulinic acid. Colonies were imaged after 22 hours of growth.

Since Mpg is required for full piliation (15), we wanted to determine whether punicalagin or chebulinic acid can access the bacterial periplasm to inhibit Mpg and reduce T4p expression on the cell surface. We used a pilus-dependent colony morphology assay to determine whether punicalagin or chebulinic acid altered *N. gonorrhoeae*-piliated colony morphology (Fig. 4A and B) (17, 39). We used a *N. gonorrhoeae* mutant strain that harbors mutations, preventing pilus antigenic and phase variation and stabilizing pili production to prevent pilus phase variation from altering the results. The *Δmpg* strain was a control for loss of Mpg activity and under-piliated colony morphology. After 22 hours of growth on solid media, treatment with 1 µM punicalagin or 1 µM chebulinic acid shifted the strain-piliated colony morphology to an under-piliated colony morphology (Fig. 4A and B). The *Δmpg* strain did not change its colony morphology when grown with 1 µM punicalagin (Fig. 4A).

**Fig 5:**
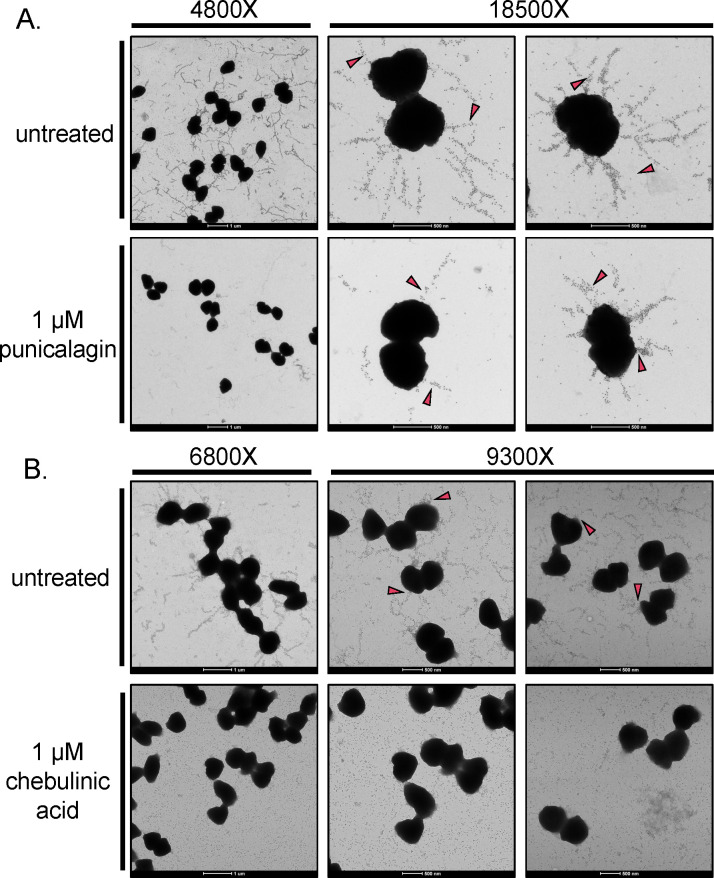
Treatment with punicalagin or chebulinic acid reduces *N. gonorrhoeae* piliation. Immunogold-TEM of pili on gonococci treated with or without (A) 1 µM punicalagin or (**B)** 1 µM chebulinic acid. Assays were performed in a strain containing a *pilE-myc* allele, and pilus expression was detected using an α-Myc primary antibody and a 5 nm gold-conjugated secondary antibody. Magenta arrows indicate pilus fibers marked with 5 nm gold particles.

To ensure colony morphology changes were due to a loss of piliation, we performed immunogold-transmission electron microscopy (immunogold-TEM) to visualize pili on the surface of individual *N. gonorrhoeae* (Fig. 5A and B). We analyzed a *N. gonorrhoeae* strain containing a carboxy-terminus myc-tagged *pilE* allele to detect pili using a monoclonal Myc antibody and a 5 nm gold-conjugated secondary antibody (40). Many pili were detected on the surface of untreated cells; however, treatment with 1 µM punicalagin or chebulinic acid reduced the number of pili on individual *N. gonorrhoeae*, similar to a *mpg* mutant (Fig. 5A and B). These data show that even though these natural compounds are complex chemical structures, they can gain access to Mpg that is localized in the periplasm to inhibit piliation.

### Treatment with punicalagin or chebulinic acid does not reduce transformation efficiency or resistance to LL-37 killing

Treatment with punicalagin and chebulinic acid decreased piliation on the cell surface. Because the pilus is involved in DNA uptake and natural transformation, we determined whether treatment of *N. gonorrhoeae* with punicalagin and chebulinic acid would inhibit transformation competence. The parental strain was a positive control for transformation, and a Δ*pilE* strain lacking the *pilE* gene was the negative control. The *mpg* mutant strain lacking the *mpg* gene was a control for a loss of Mpg activity, which we previously showed leads to reductions in transformation competence (16). Punicalagin or chebulinic acid treatment did not impact transformation efficiency in any of the tested *N. gonorrhoeae* strains relative to untreated samples (Fig. S7A).

We previously showed that the pilus promotes resistance to nonoxidative killing mediated by neutrophils through the antimicrobial peptide LL-37 (15). We wanted to determine whether punicalagin or chebulinic acid treatment led to increased sensitivity to LL-37-mediated killing. The parental strain served as a positive control for resistance to LL-37 killing, while the Δ*pilE* strain served as a negative control. Treatment with punicalagin or chebulinic acid did not alter sensitivity to LL-37 killing (Fig. S7B).

### Punicalagin and chebulinic acid decrease pilus-mediated adherence

**Fig 6:**
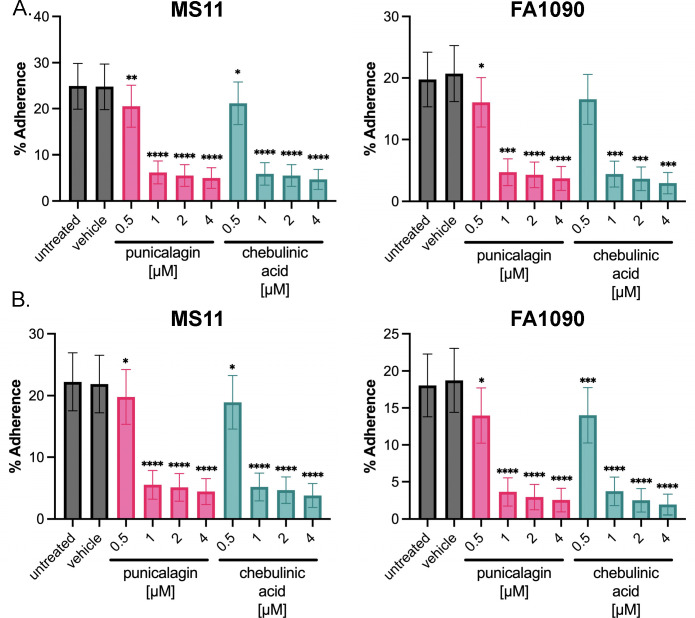
Punicalagin or chebulinic acid treatment decreases adherence of *N. gonorrhoeae* to UEC and Pex cells. *N. gonorrhoeae* pre-treated (1 hour) without or with 0.1% DMSO (vehicle) or (0.5, 1, 2, or 4 µM) punicalagin or chebulinic acid was then used to infect (**A)** Pex cells or (**B)** UECs (MOI = 100) with either *N. gonorrhoeae* strain MS11 or FA1090 for 1 hour, infection medium was removed, cells were rinsed and lysed, and serial dilutions were plated. Compounds were maintained in the medium throughout the infection. The percentage of *N. gonorrhoeae* associated with host cells was determined as a function of the untreated control (set to 100%). A nonparametric ANOVA was used to determine statistical significance. ****P < 0.0001, ***P < 0.001, **P < 0.01, and *P < 0.05.

**Fig 7:**
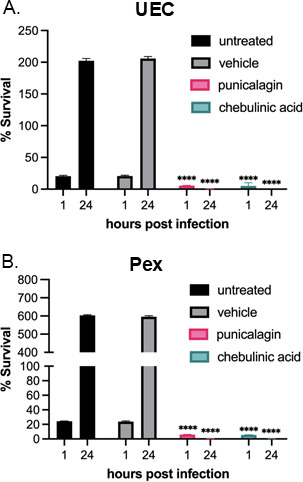
Punicalagin or chebulinic acid treatment reduces *N. gonorrhoeae* adherence and decreases survival in UEC and Pex cells. *N. gonorrhoeae* strain MS11 pre-treated (1 hour) without or with 0.1% DMSO (vehicle) or 1 µM punicalagin or chebulinic acid was used to infect (**A)** UECs or (**B)** Pex cells (MOI = 100) for 1 hour or 24 hours. At each time point, the infection medium was removed, cells were rinsed and lysed, and serial dilutions were plated. Compounds were maintained in the medium throughout the infection. The percentage of *N. gonorrhoeae* associated with host cells or that survived treatment was determined as a function of the untreated control (set to 100%). A nonparametric ANOVA was used to determine statistical significance. ****P < 0.0001.

In addition to natural transformation and resistance to nonoxidative killing by PMNs, the T4p mediates adherence to the mucosal epithelium (12–15). This adherence is the first step in colonization during infection. To determine if punicalagin and chebulinic acid treatment impairs *N. gonorrhoeae* adherence, we used cellular models of infection. We used two relevant cell types, primary male urethral cells (UECs) and cervical (Pex) epithelial cells derived from human tissues, to quantify *N. gonorrhoeae* adherence (Fig. 6) and survival during infection *in vitro* (Fig. 7). Approximately 20% of *N. gonorrhoeae* strain MS11 or FA1090 cells without treatment with punicalagin or chebulinic acid or with 0.1% DMSO (vehicle) adhered to Pex cells after 1 hour of infection (Fig. 6A). At 0.5 µM of punicalagin or chebulinic acid, both *N. gonorrhoeae* strains showed slightly reduced Pex cell adherence. However, treatment between 1 and 4 µM punicalagin or chebulinic acid significantly reduced adherence to Pex cells in both *N. gonorrhoeae* strains (Fig. 6A). Similarly, treatment with 1 to 4 µM punicalagin or chebulinic acid also significantly decreased the ability of both *N. gonorrhoeae* strains to adhere to UECs compared with the untreated, vehicle-treated, or 0.5 μΜ compound-treated samples (Fig. 6B). These data illustrate that punicalagin and chebulinic acid inhibit host-cell adherence mediated by the T4p.

We measured *N. gonorrhoeae* survival during a longer 24-hour period of infection to determine if treatment with 1 µM punicalagin or chebulinic acid prevented growth during infection following the initial colonization step. We quantified *N. gonorrhoeae* survival at 24 hours relative to the inoculum. We used untreated cells as a control for active infection and 0.1% DMSO as a vehicle control. The untreated and vehicle-treated *N. gonorrhoeae* showed growth during a 24-hour infection of UECs, with approximately 200% survival compared with the inoculum, while treatment with punicalagin or chebulinic acid resulted in almost no surviving cells (0.21% in punicalagin; 0.19% in chebulinic acid) (Fig. 7A). Similarly, punicalagin and chebulinic acid reduced *N. gonorrhoeae* survival at 24 hours in Pex cells (0.16% in punicalagin; 0.13% in chebulinic acid), while the untreated and vehicle control-treated cells showed growth during the 24-hour period, with approximately 600% survival relative to the inoculum (Fig. 7B). Therefore, punicalagin and chebulinic acid prevent *N. gonorrhoeae* survival *in vitro*.

### Punicalagin and chebulinic acid do not reduce metabolic activity or cause cytotoxicity in HeLa cells

To determine whether punicalagin and chebulinic acid negatively affect eukaryotic cell viability, we monitored cellular metabolism and cytotoxicity in HeLa cells following treatment with punicalagin or chebulinic acid. Doxorubicin, a chemotherapy drug that inhibits topoisomerase II, served as a positive control for decreasing metabolic activity (41). No change in metabolic activity was measured with punicalagin or chebulinic acid concentrations up to 5 µM (Fig. S8A). As a positive control, increasing concentrations of doxorubicin reduced the metabolic activity of HeLa cells (Fig. S8A).

In addition to cellular metabolism, we measured the cytotoxicity and viability of HeLa cells after treatment with 1 μΜ to 5 µM punicalagin or chebulinic acid. To quantify live cells, we used calcein AM, a cell-permeable compound converted to fluorescent, cell-impermeant calcein within live cells. Doxorubicin served as a positive control for cytotoxicity. Treatment with doxorubicin decreased the percentage of live cells compared with untreated cells in a dose-dependent manner. Punicalagin and chebulinic acid treatment did not decrease HeLa cell viability (Fig. S8B). We quantified dead cells with the membrane-impermeable dye ethidium homodimer (EthD-1). Cells treated with doxorubicin exhibited a dose-dependent response with higher levels of cell death than those treated with punicalagin or chebulinic acid (Fig. S8C).

Using UEC and Pex cells, we quantified viability of primary cells under treatment with punicalagin or chebulinic acid. We used a fluorometric blue assay kit to determine cell viability in the presence of increasing concentrations of punicalagin or chebulinic acid (Fig. S9). The 0.1% DMSO vehicle control showed no decrease in cell viability in either cell line. In concentrations from 0.5 to 4 µM punicalagin or chebulinic acid, there was no significant decrease in cell viability in UECs or Pex cells (Fig. S9). There were small, significantly different decreases in cell viability at 8 µM concentrations of either natural compound. These data suggest that these natural compounds do not impact viability of UECs or Pex cells at lower micromolar concentrations.

## DISCUSSION

Mpg is established as a M23B zinc metallopeptidase important for *N. gonorrhoeae* piliation and pilus function. Here, we used a novel high-throughput screen to identify two natural compounds, punicalagin and chebulinic acid, which bind and inhibit Mpg activity. Punicalagin and chebulinic acid are naturally occurring phenolic compounds in the ellagitannin family (42, 43). Both compounds have been studied previously in other contexts, and in mammalian cells, these compounds target many different enzymes (33, 44). Punicalagin and its derivatives have activity against various bacterial species, including *Clostridioides difficile*, *S. aureus*, *Salmonella enterica* serovar Typhi, serovar Typhimurium, and *E. coli* (45–48). Moreover, punicalagin targets *S. aureus* sortase A, preventing proper localization of proteins required for interactions with host cells during infection (47). Chebulinic acid also exhibits activity against *H. pylori* and *A. baumannii*, but the mechanism of action was not determined (49, 50). Chebulinic acid is predicted to interact with the CagA protein in *H. pylori*, preventing CagA from interacting with host cells to initiate infection (49). Additionally, chebulinic acid has demonstrated antiviral activity against herpes simplex virus-2, dengue, and chikungunya viral infection (51, 52). Chebulinic acid inhibited the production of various viral glycoproteins, including the major envelope protein of dengue virus (52). The wide range of anti-microbial activities suggests these compounds may have many anti-microbials roles.

Using molecular docking studies, we determined punicalagin could bind Mpg at three sites that reside in the LysM and LytM domains. Site 1 binding would disrupt the Zn^2+^ binding within the active site, while Site 2 and Site 3 binding could disrupt PG chain binding. Blocking any of these sites could reduce Mpg PG-hydrolyzing activity. Our molecular docking data of Site 1 suggest that punicalagin and chebulinic acid both could block the region containing Zn^2+^ through two aromatic rings inserted into the pocket itself and the groove distal to the Zn^2+^ pocket. Therefore, these two compounds may work similarly to prevent Mpg PG-hydrolyzing activity. However, this also raises the possibility that the aromatic ring structures of these compounds could be used as scaffolds to design and test new inhibitors of Mpg or to identify additional natural compounds with similar structures. Structural or mutational studies will be necessary to determine where these compounds interact with Mpg

However, since punicalagin and chebulinic acid can inhibit the activity of many Mpg orthologs, we predict that the compounds act on Site 1. Gram-negative Mpg orthologs from *P. aeruginosa*, *A. baumannii*, and *V. cholerae* are inhibited by treatment with punicalagin or chebulinic acid, confirming that the compounds target the conserved C-terminal LytM domain of Mpg (Fig. S3; Fig. S4). The LytM domain harbors the Zn^2+^ binding pocket, which we labeled binding Site 1 in Mpg. Thus, we predict that punicalagin and chebulinic acid likely block Mpg activity through direct binding to the active site in the LytM domain, rather than blocking PG binding within Site 2 or Site 3. This loss of enzymatic activity suggests that inhibiting the activity of Mpg orthologs may have applications as M23B metalloproteases control many vital processes.

We previously showed that the PG hydrolase activity of Mpg is required for full piliation of *N. gonorrhoeae* (15). At 1 µM concentrations, inhibition of Mpg activity by treatment with punicalagin or chebulinic acid reduced pili on the *N. gonorrhoeae* cell surface, illustrated by under-piliated colony morphology and loss of surface-associated pili by TEM. Our data show that punicalagin and chebulinic acid target and inhibit *N. gonorrhoeae* piliation at a single-cell and population level. Importantly, because Mpg is periplasmic, these data illustrate that punicalagin and chebulinic acid can breach the outer membrane. Determining what portions of the compounds allow access to the periplasm could influence anti-microbial drug development.

Because exposure to punicalagin or chebulinic acid resulted in a loss of piliation, we examined other pilus-related phenotypes in the presence of these compounds. The pilus is important for natural transformation, where it is involved in DNA uptake for competence. Additionally, the pilus promotes resistance to nonoxidative killing by the neutrophil-secreted antimicrobial peptide LL-37 (15). Interestingly, treatment with punicalagin or chebulinic acid did not impact transformation efficiency or resistance to LL-37 nonoxidative killing. Punicalagin and chebulinic acid target Mpg enzymatic activity, and loss of enzymatic activity may be insufficient to reduce transformability of cells. We previously showed that low levels of PilE protein are sufficient to maintain transformation competence (53), which may explain why treatment with punicalagin or chebulinic acid does not reduce transformation efficiency.

*N. gonorrhoeae* requires the T4p for initial colonization, where the T4p mediates adherence to the mucosal epithelium (12, 14). We showed that punicalagin and chebulinic acid can significantly reduce adherence to infection-relevant cell types using human primary male UECs and Pex epithelial cells. Inhibition of adherence by punicalagin and chebulinic acid occurs at as low as 1 µM dosage. Moreover, treatment with punicalagin or chebulinic acid further prevents survival at longer infection times of 24 hours, where less than 1% of the total inoculum is recovered from UECs or Pex cell infections. These data support a role for these natural compounds in preventing colonization and pathogenesis mediated by the T4p.

We showed that neither punicalagin nor chebulinic acid had cytotoxic effects on primary and immortalized host cells at concentrations up to 5 µM and this has been shown previously in other cell culture models (49, 52, 54). Previous work has shown these compounds are not generally toxic in mice or rats (55, 56). However, these compounds have short half-lives in animal models and punicalagin is not stable as it is readily catabolized (44, 57, 58). Interestingly, previous work has illustrated that urolithin A, a punicalagin breakdown product, shows activity against *C. difficile* (45). We tested the efficacy of punicalagin catabolic derivatives against Mpg and found that in combination, some breakdown products exhibit activity against Mpg. This result illustrates that punicalagin, even when metabolized, could potentially serve as a therapeutic compound to target *N. gonorrhoeae* during infection. While our data show that punicalagin and chebulinic acid prevent adherence and survival in cell culture models of infection, future work will be needed to determine if punicalagin or chebulinic acid can prevent resistance to PMN killing.

We wanted to determine if these compounds alter the activity of or resistance to the current first-line recommended antibiotic for gonorrhea, ceftriaxone, when used synergistically. Using antibiotic E-tests, we measured the resistance of *N. gonorrhoeae* strain MS11 to ceftriaxone in the presence or absence of 1 µM punicalagin or chebulinic acid. These natural compounds had no effect on sensitivity to ceftriaxone, where sensitivity under all conditions occurred at 0.006 µg/mL. Thus, these natural compounds may have the potential to be used in conjunction with ceftriaxone. However, this needs to be further investigated to determine if there is an added benefit of using natural compounds and ceftriaxone concomitantly in the context of colonization and infection through the additive action of the antibiotic and the loss of pilus-dependent abilities by use of natural compounds.

Our findings provide a proof-of-concept that we can employ HTS approaches in combination with target-based screens to identify inhibitors of specific T4p components in *N. gonorrhoeae* selectively. Moreover, we identify two natural compounds with activity against the Mpg that decrease *N. gonorrhoeae* piliation and reduce adherence and survival *in vitro*. Since these compounds are also active against other M23B metallopeptidases, they may affect piliation and other bacterial processes that are important for growth or pathogenesis. Given the importance of the T4p across stages of *N. gonorrhoeae* pathogenesis, the activity of these natural compounds against the T4p suggests these types of compounds may be used to target multiple points of *N. gonorrhoeae* infection. As punicalagin and chebulinic acid are not stable in animals, they will not be viable treatments for gonorrhea infections. However, our work with punicalagin and chebulinic acid may lead to the development of other viable anti-virulence and anti-colonization compounds.

## MATERIALS AND METHODS

### Bacterial strains and media

All gonococcal strains were derivatives of the FA1090 strain N-1-60 (40). In the N-1-60 strain, the guanine quadruplex site upstream of the *pilE* gene is mutated to prevent antigenic variation and the *pilC1* allele is locked in an “on” conformation to ensure expression of pili (40). Detailed strain information is listed in Table S2. Gonococcal strains were grown on Gc medium base (GCB; Difco) (36.25 g/L), agar (1.25 g/L), Kellogg Supplement I (22.2mM glucose, 0.68 mM glutamine, and 0.45 mM cocarboxylase), and Kellogg Supplement II (1.23 mM Fe(NO_3_)_3_) (17, 59). Compounds for analysis were added to medium prior to solidification at concentrations indicated in the text. *N. gonorrhoeae* was grown on GCB agar with or without compounds in a 24-well plate at 37°C and 5% CO_2_ for 22 hours.

### Generation of DNA constructs

All DNA constructs were generated using oligonucleotide primers purchased from Integrated DNA Technologies (Coralville, IA). All resulting constructs were verified using targeted Sanger DNA sequencing from GeneWiz. All constructs and oligonucleotides are listed in Table S3.

### Expression constructs

The *mpg* gene and its orthologs were PCR amplified (without the predicted signal sequence) from chromosomal DNA of relative species using a forward primer with an NheI site and a reverse primer with a HindIII site, respectively (Table S3). The PCR fragment was cut by NheI/HindIII restriction enzymes and cloned into an NheI/HindIII digested pET28a overexpression vector to yield a 6× His-tagged Mpg ortholog.

### Mpg expression and purification

To purify the 6X His-Mpg ortholog proteins, *E. coli* BL21(DE3) cells (Novagen) harboring the pET28a construct containing the *mpg* gene were inoculated into 4 mL Luria Broth (LB) medium containing kanamycin (50 mg/mL), grown overnight at 37°C. These cultures were then 50× diluted into fresh medium containing kanamycin (35 mg/mL), grown another 2–3 hours (Optical Density at 600 nm [OD_600_] 0.4–0.5), and induced by the addition of 1 mM IPTG for 4 hours at 37°C. The cells were pelleted by centrifuge, resuspended in cold 1× His-Bind Buffer (Kit; Novagen) 20 mL per 100 mL volume of cell culture (Novagen), and sonicated on ice using a Vibra-Cell VC250 with a microtip (Sonics & Materials) at 35% duty cycle for a total of 3–4 minutes in pulsed mode, followed by centrifugation (15,000 × *g*) for 20 minutes to clarify the protein extract. The extract was purified on the Novagen Ni-NTA His-Bind Resin using column chromatography methods according to the manufacturer’s protocol (TB054 Rev. F0106). The elution containing purified His-Mpg orthologs was dialyzed against sodium acetate buffer (20 mM, pH 4.5, 10% glycerol) and concentrated using a 10 kDa MWCO Amicon Ultra Centrifugal Filter at 4°C.

### Northwestern high-throughput analysis laboratory compound library

37,760 molecules were screened from the Northwestern High Throughput Analysis Laboratory’s compound library: 1,280 molecules from the protein-protein interaction inhibitors collection, 11,520 from the kinase inhibitors collection (ChemDiv Inc., California), 20,480 diverse chemical structures collection (ChemBridge Corp., California), 2,880 bioactive compounds (TargetMol Chemicals Inc., Massachusetts), and 1600 compounds from the cell cycle/DNA damage collection (MedChemExpress USA, New Jersey).

### FTS assay

Recombinant Mpg showed consistent performance in the FTS assays, with a standard deviation of 0.12°C across 32 replicates and a T_m_ of 57°C. The assay was conducted in 384-well microplates, with Mpg (1 µM or 0.04 µg/µL) premixed with a 5× concentration of Sypro-Orange in HEPES buffer (20 mM HEPES, 150 mM NaCl, pH 7.5). Ten or 5 µL of the protein-dye mixture was dispensed into each well, and 10 to 50 nanoliters of compounds (10–50 μM, prepared from 10 mM stock solutions) was added using an Echo550 acoustic transfer robot (Labcyte, California). After sealing with an optical tape, centrifuging, and mixing, the thermal scan was performed from 20°C to 90°C at a ramp rate of 0.5°C/minute, with fluorescence data collected on a CFX384 real-time PCR machine (Bio-Rad Laboratories).

### Peptidoglycan isolation and labeling

PG was purified from *N. gonorrhoeae* and labeled with Remazol brilliant blue (RBB, Sigma) as described previously (16). FITC (Fluorescein Isothiocyanate Isomer 1, Sigma) labeled PG was prepared by incubating 10 mg PG with 50 mL FITC (1 mg/mL in DMSO) in 1 mL carbonate-bicarbonate buffer (0.05 M pH 9.6) at 37°C for 4 hours in the dark. The reaction was micro-centrifuged at 14,000 rpm for 10 minutes to remove the supernatant. The FITC-labeled PG pellet was washed 2× with carbonate-bicarbonate buffer, 2× with ethanol, and 6× with distilled water and finally resuspended in water containing 0.02% sodium azide and stored at 4°C in the dark.

### Dye release assay for PG hydrolysis

Ten milligrams per milliliter of RBB-PG, 1 or 2 mM purified Mpg, and compounds at indicated concentrations were incubated for 4 hours at 37°C in a total of 15 mL of assay buffer (25 mM Tris-HCl [pH7.4], 100 mM NaCl, and 0.5% Triton X100). The reaction was terminated by heating at 95°C for 5 minutes and then spun at 14,000 rpm for 10 minutes in a microcentrifuge at room temperature. Ten milliliters of supernatant was collected, and its absorbance at 595 nm was measured with a NanoDrop spectrophotometer.

### Bioinformatics

Sequences were aligned by multiple sequence alignment with hierarchical clustering using MultAlin (60). Alignments were visualized using ESPript 3 (61).

### Imaging of pilus-dependent colony morphology

*N. gonorrhoeae* colonies grown for 22 hours on solid medium were observed, and photos were taken using a Nikon SMZ-10A stereomicroscope and a Nikon digital-site camera.

### Pilus detection

For analysis of compound effect on the piliation of gonococcal strain on solid medium, immunoelectron microscopy was performed as described previously (40). Grids were viewed using an FEI Tecnai Spirit G2 TEM.

### Transformation efficiency assays

Transformation assays were performed similarly as previously published, with modifications (53). *N. gonorrhoeae* was struck from frozen stocks and grown overnight on GCB plates at 37°C under 5% CO_2_. Individual colonies were picked and struck on GCB with no compound, 1 µM punicalagin, or 1 µM chebulinic acid. Colonies were grown overnight at 37°C under 5% CO_2_. *N. gonorrhoeae* was collected in 400 µL of Gonococcal Medium Base-Liquid (GCBL) (supplemented with Kellogg Supplement I and II and 5 mM MgSO_4_) with no compound, 1 µM punicalagin, or 1 µM chebulinic acid using a polyester swab. Cultures were diluted to an OD_550_ of 0.15 in a 200 µL total volume of GCBL with no compound, 1 µM punicalagin, or 1 µM chebulinic acid. Two microliters of a 25 ng/µL pSY6 plasmid (62) DNA stock was added to each 200 µL reaction and incubated at 37°C for 20 minutes. Then, 1 U of DNase I was added to each tube and incubated at 37°C for 10 minutes. For each reaction, the entire reaction was then added to 2 mL of 37°C prewarmed GCBL media with no compound, 1 µM punicalagin, or 1 µM chebulinic acid in a 24-well plate and incubated at 37°C under 5% CO_2_ for 2 hours. These 2.2 mL reactions were mixed, and a portion was harvested for 10-fold serial dilutions for CFU plating. Serial dilutions were performed using GCBL without punicalagin or chebulinic acid. Ten microliters of each serial dilution from 10^0^ to 10^−6^ was plated on both GCB and GCB containing 1 µg/mL nalidixic acid. CFU were counted using a stereoscope after overnight growth at 37°C under 5% CO_2_.

### LL-37 sensitivity assays

LL-37 sensitivity assays were performed as described previously (15). Briefly, cells were grown on GCB plates supplemented with Kellogg Supplement I and II with or without punicalagin or chebulinic acid. Cells were harvested using a polyester swab and resuspended in GCBL (supplemented with Kellogg Supplement I and 50 mM sodium bicarbonate) to an OD_550_ between 0.03 and 0.05. Cultures were grown for 2 hours at 37°C with shaking (220 rpm). Cells were diluted to an OD_550_ of 0.05 in 0.5 mL of GCBL (with Supplement I and sodium bicarbonate). Cells were grown in the presence or absence of 1 µM LL-37 (Peptide Sciences) for 20 minutes at 37°C with shaking. Cultures were centrifuged for 2 minutes at 4,000 rpm, and 440 µL of supernatant was removed. Cells were resuspended in 440 µL of GCBL, and 10-fold serial dilutions were plated on GCB agar plates. Relative survival is the ratio of LL-37-resistant colonies relative to the CFU of cells in the absence of LL-37.

### Antibiotic sensitivity E-tests

Overnight colonies of *N. gonorrhoeae* strain MS11 were resuspended in fresh GCB (with Kellogg Supplement I and II) and adjusted to an OD_600_ of 0.1. Two dips of a polyester swab into the cell resuspension were applied to the entire surface of a GCB plate with or without 1 µM of punicalagin or chebulinic acid. Liofilchem ceftriaxone MTS MIC Test Strips (0.002 μg/mL–32 μg/mL) were placed in the center of the plates. Plates were incubated at 37°C under 5% CO_2_ for 20 hours.

### UEC and Pex cell cultures

Primary male UECs and Pex epithelial cells were procured from de-identified human tissues, as described (63, 64). In brief, this process involves the outgrowth of epithelial cells from dissected urethral or cervical tissue. Urethral tissue was obtained from the National Disease Research Interchange (Philadelphia, PA, USA), whereas cervical tissue was obtained from the Cooperative Human Tissue Network (Columbus, OH, USA). Tissue explants and primary epithelial cells were maintained using human urethral epithelial cell media (Cell Applications, San Diego, CA, USA) for UECs or defined keratinocyte serum-free medium (dk-SFM; Gibco, Grand Island, NY, USA) for Pex cells. The use of these tissues does not constitute human subject research, as determined by the Institutional Review Board at the Abigail Wexner Research Institute at Nationwide Children’s Hospital.

### UEC and Pex host cell viability assays

To determine if punicalagin or chebulinic acid exerted a cytotoxic effect toward uninfected UECs or Pex cells, cells were incubated for 24 hours with 0.1% DMSO (vehicle), 0.5, 1, 2, 4, or 8 µM of each drug, or they were left untreated. UEC and Pex cell viability was determined fluorometrically using the Cell Viability Assay Kit (Fluorometric-Blue), according to the manufacturer’s instructions (Abcam, Waltham, MA, USA). Assays were performed in duplicate on three separate occasions. Data were adjusted for background after which fluorescence recorded for each condition was normalized to fluorescence recorded for untreated cells. Paired Student’s *t*-test (GraphPad version 8.2.0 for MacOs, GraphPad Software, San Diego, California, USA) was used to determine the statistical significance of data obtained.

### UEC and Pex cell infection assays

Infection assays were performed by pre-incubating (1 hour, 37°C) bacteria with medium alone or with medium supplemented with 0.1% DMSO (vehicle) or 0.5, 1, 2, or 4 µM punicalagin or chebulinic acid, as noted, for 1 hour before infection of each host cell type. For association assays, bacteria were incubated (37°C) with host cells for 1 hour following pre-treatment with each drug, whereas survival assays comprised of a 24-hour incubation. DMSO (vehicle control), punicalagin, or chebulinic acid were maintained in the medium throughout the course of infection. For both assays, host cell monolayers were rinsed following infection and lysed. Serial dilutions of the host cell lysates were plated, and viable gonococci were enumerated by counting CFUs after a 48-hour incubation (37°C, 5% CO_2_). The percentage of *N. gonorrhoeae* that associated with host cells or that survived treatment was determined as a function of the untreated control (set to 100%). All assays were performed in triplicate on three separate occasions using a multiplicity of infection of 100. A nonparametric ANOVA was used to determine the statistical significance of bacterial association or survival (GraphPad).

### HeLa cell culture

HeLa cells were cultured in Dulbecco’s modified Eagle’s medium (DMEM) (Gibco, Dublin, Ireland) with 10% heat-inactivated fetal bovine serum (FBS) (VWR, Radnor, PA). Cells were washed with 1× sterile phosphate-buffered saline (PBS) (Gibco) and harvested using 0.25% trypsin-EDTA digestion (Gibco). All experiments were performed at 37°C with 5% CO_2_.

### HeLa MTS assays

HeLa cells were seeded in 100 µL DMEM plus 10% FBS per well at 2 × 10^4^ cells/well in a 96-well plate (Greiner Bio-One, Germany) and allowed to grow for 24 hours. Media were removed, cells were washed, and 80 µL of fresh media was added. Compounds were added in a 20 µL volume to final concentrations of 1, 2, 3, or 5 μΜ in technical triplicate. Treatment was allowed to proceed for 24 hours before media were removed, and cells were washed three times with 1× PBS. One hundred microliters of 1× PBS was added to each well. Twenty microliters of MTS reagent (CellTiter 96 AQ_ueous_ One Solution Cell Proliferation Assay, Promega) was added to each well. Cells were incubated with MTS reagent for 1 hour at 37°C under 5% CO_2_ before absorbance (A_490_) was read on a Spectramax M5 spectrophotometer. Assays were performed in biological triplicate.

### HeLa live/dead viability/cytotoxicity assays

HeLa cells were seeded in 100 µL DMEM plus 10% FBS per well at 2 × 10^4^ cells/well in a 96-well plate (Greiner Bio-One, Germany) and grown for 24 hours at 37°C under 5% CO_2_. Media were removed, cells were washed twice with 1× PBS, and 80 µL of fresh media was added. Compounds were added in a 20 µL volume to final concentrations of 1, 2, 3, or 5 μΜ in technical triplicate. Treatment was allowed to proceed for 24 hours before media were removed and cells were washed three times with 1× PBS. To assay viability/cytotoxicity, PBS was aspirated and 100 µL of an EthD-1 (4 µM) and Calcein AM (2 µM) solution (live/dead viability/cytotoxicity kit; Life Technologies, Carlsbad, CA) in 1× PBS was added. Cells were incubated for 30 minutes before fluorescence was measured (645 nm excitation/530 nm emission) on a Spectramax M5 plate reader. Assays were performed in biological triplicate.

### Statistical analysis

All statistical analyses were performed in GraphPad Prism 9 version 9.4.1. Statistical significance was determined by performing an ordinary one-way ANOVA followed by a Dunnett’s multiple comparisons test or two-way ANOVA. *P* values for individual experiments are noted in figure legends and in the text. All experiments were conducted on at least two biological replicates, each with technical triplicates where available.

## SUPPLEMENTAL MATERIAL

Supplemental Figures mbio.04027-24-s0001.pdf Figures S1 to S9.

Supplemental Tables mbio.04027-24-s0002.docx Tables S1 to S4.

ASM does not own the copyrights to Supplemental Material that may be linked to, or accessed through, an article. The authors have granted ASM a non-exclusive, world-wide license to publish the Supplemental Material files. Please contact the corresponding author directly for reuse.
