# Supplementary material for: Natural compounds target the M23B zinc metallopeptidase Mpg to modulate Neisseria gonorrhoeae Type IV pilus expression
Source: mBio. 2025 Feb 25;16(4):e04027-24. doi: 10.1128/mbio.04027-24 (PMC11980366; doi:10.1128/mbio.04027-24)
Supplement: Supplemental Figures — Figures S1 to S9. [file mbio.04027-24-s0001.pdf]

Fig. S1

A.

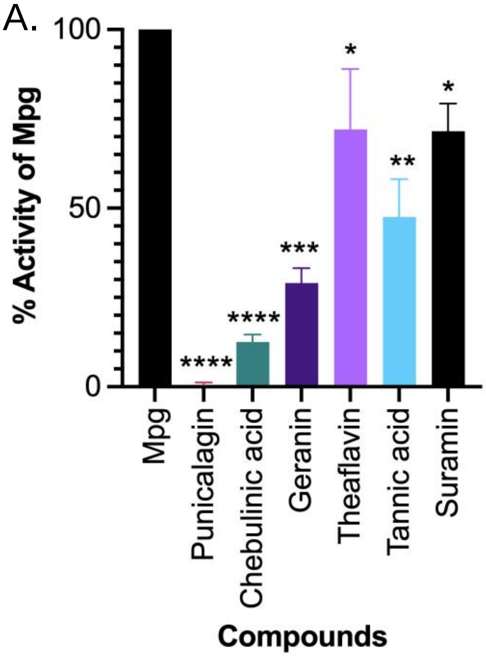

100
50
0
% Activity of Mpg
*
*
**
***
****
****
Mpg
Punicalagin
Chebulinic acid
Geranin
Theaflavin
Tannic acid
Suramin
Compounds

B.

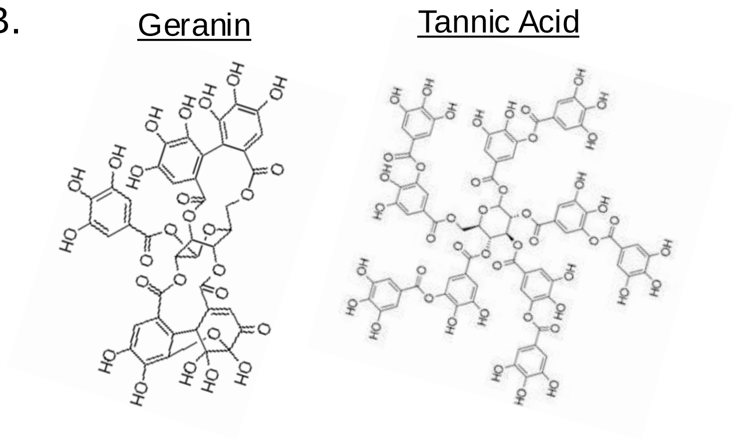

Geranin
Tannic Acid

Fig. S2

A.

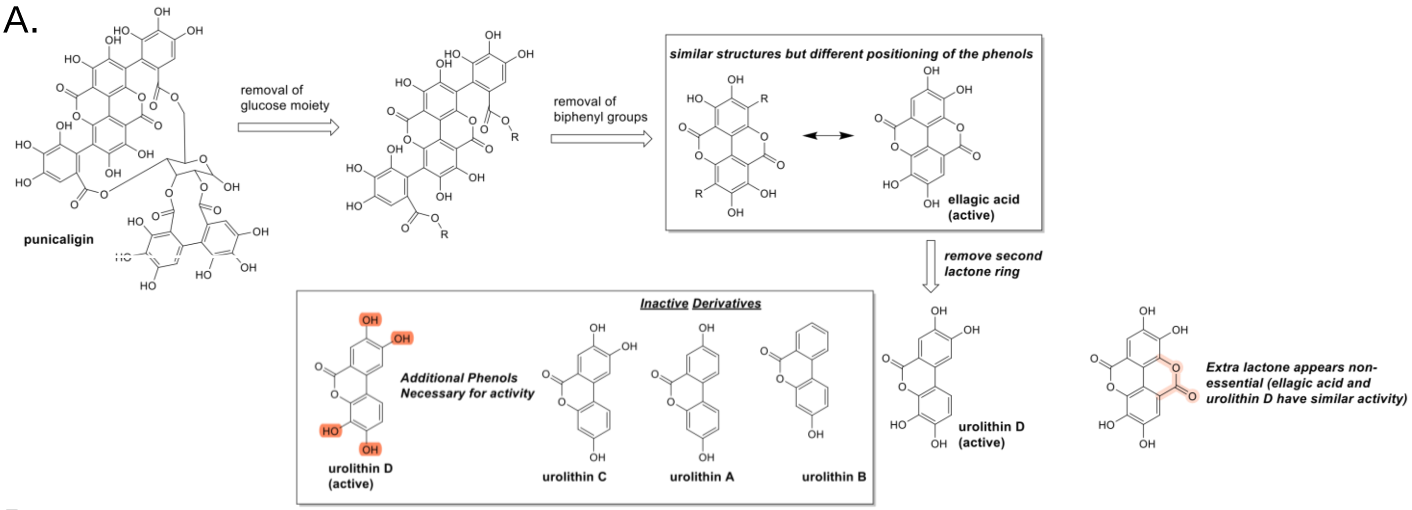

removal of glucose moiety
removal of biphenyl groups
similar structures but different positioning of the phenols
punicaligin
ellagic acid (active)
remove second lactone ring
Inactive Derivatives
Additional Phenols Necessary for activity
urolithin D (active)
urolithin C
urolithin A
urolithin B
urolithin D (active)
Extra lactone appears non-essential (ellagic acid and urolithin D have similar activity)

B.

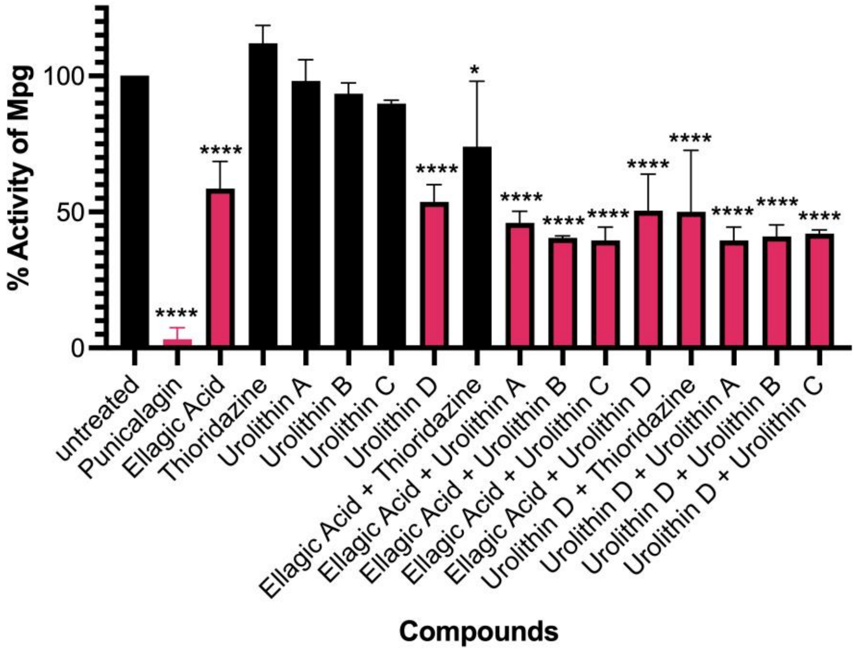

% Activity of Mpg
100
50
0
****
*
untreated
Punicalagin
Ellagic Acid
Thioridazine
Urolithin A
Urolithin B
Urolithin C
Urolithin D
Ellagic Acid + Thioridazine
Ellagic Acid + Urolithin A
Ellagic Acid + Urolithin B
Ellagic Acid + Urolithin C
Ellagic Acid + Urolithin D
Urolithin D + Thioridazine
Urolithin D + Urolithin A
Urolithin D + Urolithin B
Urolithin D + Urolithin C
Compounds

Fig. S3

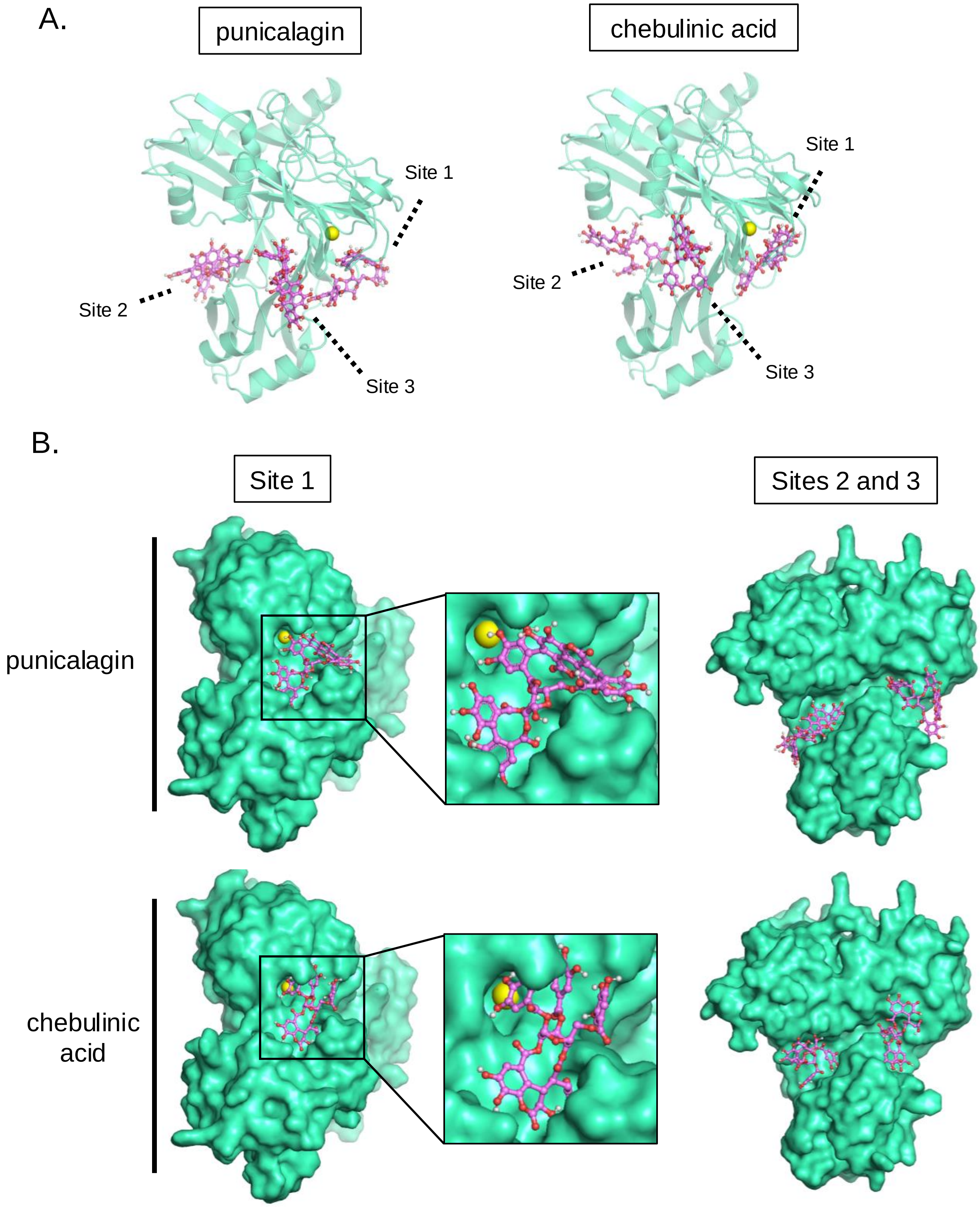
A.
punicalagin
chebulinic acid
Site 1
Site 2
Site 3
Site 1
Site 2
Site 3
B.
Site 1
Sites 2 and 3
punicalagin
chebulinic acid

Fig. S4

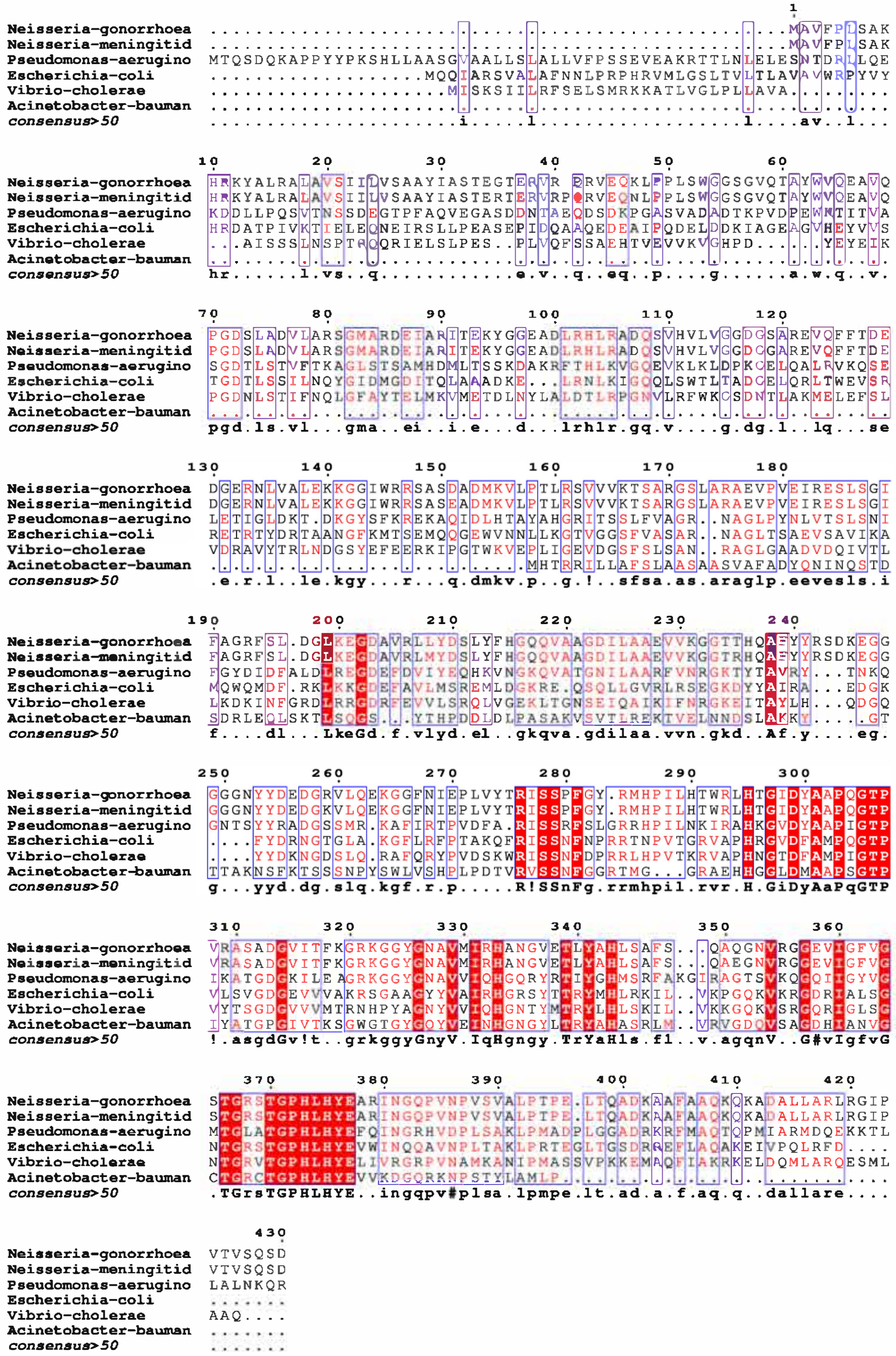
1
Neisseria-gonorrhoea ....MAVFPLSAK
Neisseria-meningitid ....MAVFPLSAK
Pseudomonas-aerugino MTQSDQKAPPYYPKSHLLAASGVAALLSLALLVFPSSEVEAKRTTLNLELESNTDRLLQE
Escherichia-coli ...................MQQIARSVALAFNNLPRPHRVMLGSLTVLTLAVAVWRPYVY
Vibrio-cholerae ......................MISKSIILRFSELSMRKKATLVGLPLLAVA........
Acinetobacter-bauman ....
consensus>50 ....i.....l..................l....av..l...
10 20 30 40 50 60
Neisseria-gonorrhoea HRKYALRALAVSIILVSAAYIASTEGTERVR PRVEQKLPPLSWGGSGVQTAYWVQEAVQ
Neisseria-meningitid HRKYALRALAVSIILVSAAYIASTERTERVRP●RVEQNLPPLSWGGSGVQTAYWVQEAVQ
Pseudomonas-aerugino KDDLLPQSVTNSSDEGTPFAQVEGASDDNTAEQDSDKPGASVADADTKPVDPEWKTITVA
Escherichia-coli HRDATPIVKTIELEQNEIRSLLPEASEPIDQAAQEDEAIPQDELDDKIAGEAGVHEYVVS
Vibrio-cholerae ...AISSSLNSPTAQQRIELSLPES..PLVQFSSAEHTVEVVKVGHPD......YEYEIK
Acinetobacter-bauman ....
consensus>50 hr......l.vs..q............e.v..q..eq..p....g......a.w.q..v.
70 80 90 100 110 120
Neisseria-gonorrhoea PGDSLADVLARSGMARDEIARITEKYGGEADLRHLRADQSVHVLVGGDGSAREVQFFTDE
Neisseria-meningitid PGDSLADVLARSGMARDEIARITEKYGGEADLRHLRADQSVHVLVGGDCGAREVQFFTDE
Pseudomonas-aerugino SGDTLSTVFTKAGLSTSAMHDMLTSSKDAKRFTHLKVGQEVKLKLDPKCELQALRVKQSE
Escherichia-coli TGDTLSSILNQYGIDMGDITQLAAADKE...LRNLKIGQQLSWTLTADCELQRLTWEVSR
Vibrio-cholerae PGDNLSTIFNQLGFAYTELMKVMETDLNYLALDTLRPGNVLRFWKCSDNTLAKMELEFSL
Acinetobacter-bauman ....
consensus>50 pgd.ls.vl...gma..ei..i.e...d...lrhlr.gq.v....g.dg.l..lq...se
130 140 150 160 170 180
Neisseria-gonorrhoea DGERNLVALEKKGGIWRRSASDADMKVLPTLRSVVVKTSARGSLARAEVPVEIRESLSGI
Neisseria-meningitid DGERNLVALEKKGGIWRRSASEADMKVLPTLRSVVVKTSARGSLARAEVPVEIRESLSGI
Pseudomonas-aerugino LETIGLDKT.DKGYSFKREKAQIDLHTAYAHGRITSSLFVAGR..NAGLPYNLVTSLSNI
Escherichia-coli RETRTYDRTAANGFKMTSEMQQGEWVNNLLKGTVGGSFVASAR..NAGLTSAEVSAVIKA
Vibrio-cholerae VDRAVYTRLNDGSYEFEERKIPGTWKVEPLIGEVDGSFSLSAN..RAGLGAADVDQIVTL
Acinetobacter-bauman ............................MHTRRILLAFSLAASAASVAFADYQNINQSTD
consensus>50 .e.r.l..le.kgy...r...q.dmkv.p..g.!..sfsa.as.araglp.eevesls.i
190 200 210 220 230 240
Neisseria-gonorrhoea FAGRFSL.DGLKEGDAVRLLYDSLYFHGQQVAAGDILAAEVVKGGTTHQAFYYRSDKEGG
Neisseria-meningitid FAGRFSL.DGLKEGDAVRLMYDSLYFHGQQVAAGDILAAEVVKGGTRHQAFYYRSDKEGG
Pseudomonas-aerugino FGYDIDFALDLREGDEFDVIYEQHKVNGKQVATGNILAARFVNRGKTYTAVRY...TNKQ
Escherichia-coli MQWQMDF.RKLKKGDEFAVLMSREMLDGKRE.QSQLLGVRLRSEGKDYYAIRA...EDGK
Vibrio-cholerae LKDKINFGRDLRRGDRFEVVLSRQLVGEKLTGNSEIQAIKIFNRGKEITAYLH...QDGQ
Acinetobacter-bauman SDRLEQLSKTLSQGS...YTHPDDLDLPASAKVSVTLREKTVELNNDSLAKKY.....GT
consensus>50 f....dl...LkeGd.f.vlyd.el..gkqva.gdilaa.vvn.gkd..Af.y....eg.
250 260 270 280 290 300
Neisseria-gonorrhoea GGGNYYDEDGRVLQEKGGFNIEPLVYTRISSPFGY.RMHPILHTWRLHTGIDYAAPQGTP
Neisseria-meningitid GGGNYYDEDGKVLQEKGGFNIEPLVYTRISSPFGY.RMHPILHTWRLHTGIDYAAPQGTP
Pseudomonas-aerugino GNTSYYRADGSSMR.KAFIRTPVDFA.RISSRFSLGRRHPILNKIRAHKGVDYAAPIGTP
Escherichia-coli ....FYDRNGTGLA.KGFLRFPTAKQFRISSNFNPRRTNPVTGRVAPHRGVDFAMPQGTP
Vibrio-cholerae ....YYDKNGDSLQ.RAFQRYPVDSKWRISSNFDPRRLHPVTKRVAPHNGTDFAMPIGTP
Acinetobacter-bauman TTAKNSFKTSSSNPYSWLVSHPLPDTVRVSSNFGGRTMG...GRAEHHGGLDMAAPSGTP
consensus>50 g...yyd.dg.slq.kgf.r.p.....R!SSnFg.rrmhpil.rvr.H.GiDyAaPqGTP
310 320 330 340 350 360
Neisseria-gonorrhoea VRASADGVITFKGRKGGYGNAVMIRHANGVETLYAHLSAFS...QAQGNVRGGEVIGFVG
Neisseria-meningitid VRASADGVITFKGRKGGYGNAVMIRHANGVETLYAHLSAFS...QAEGNVRGGEVIGFVG
Pseudomonas-aerugino IKATGDGKILEAGRKGGYGNAVVIQHGQRYRTIYGHMSRFAKGIRAGTSVKQGQIIGYVG
Escherichia-coli VLSVGDGEVVVAKRSGAAGYYVAIRHGRSYTTRYMHLRKIL..VKPGQKVKRGDRIALSG
Vibrio-cholerae VYTSGDGVVVMTRNHPYAGNYVVIQHGNTYMTRYLHLSKIL..VKKGQKVSRGQRIGLSG
Acinetobacter-bauman IYATGPGIVTKSGWGTGYGQYVEINHGNGYLTRYAHASRLM..VRVGDQVSAGDHIANVG
consensus>50 !.asgdGv!t..grkggyGnyV.IqHgngy.TrYaHls.fl..v.agqnV..G#vIgfvG
370 380 390 400 410 420
Neisseria-gonorrhoea STGRSTGPHLHYEARINGQPVNPVSVALPTPE.LTQADKAAFAAQKQKADALLARLRGIP
Neisseria-meningitid STGRSTGPHLHYEARINGQPVNPVSVALPTPE.LTQADKAAFAAQKQKADALLARLRGIP
Pseudomonas-aerugino MTGLATGPHLHYEFQINGRHVDPLSAKLPMADPLGGADRKRFMAQTQPMIARMDQEKKTL
Escherichia-coli NTGRSTGPHLHYEVWINQQAVNPLTAKLPRTEGLTGSDRQEFLAQAKEIVPQLRFD....
Vibrio-cholerae NTGRVTGPHLHYELIVRGRPVNAMKANIPMASSVPKKEMAQFIAKRKELDQMLARQESML
Acinetobacter-bauman CTGRCTGPHLHYEVVKDGQRKNPSTYLAMLP.............................
consensus>50 .TGrsTGPHLHYE..ingqpv#plsa.lpmpe.lt.ad.a.f.aq.q..dallare....
430
Neisseria-gonorrhoea VTVSQSD
Neisseria-meningitid VTVSQSD
Pseudomonas-aerugino LALNKQR
Escherichia-coli ....
Vibrio-cholerae AAQ....
Acinetobacter-bauman ....
consensus>50 ....

Fig. S5

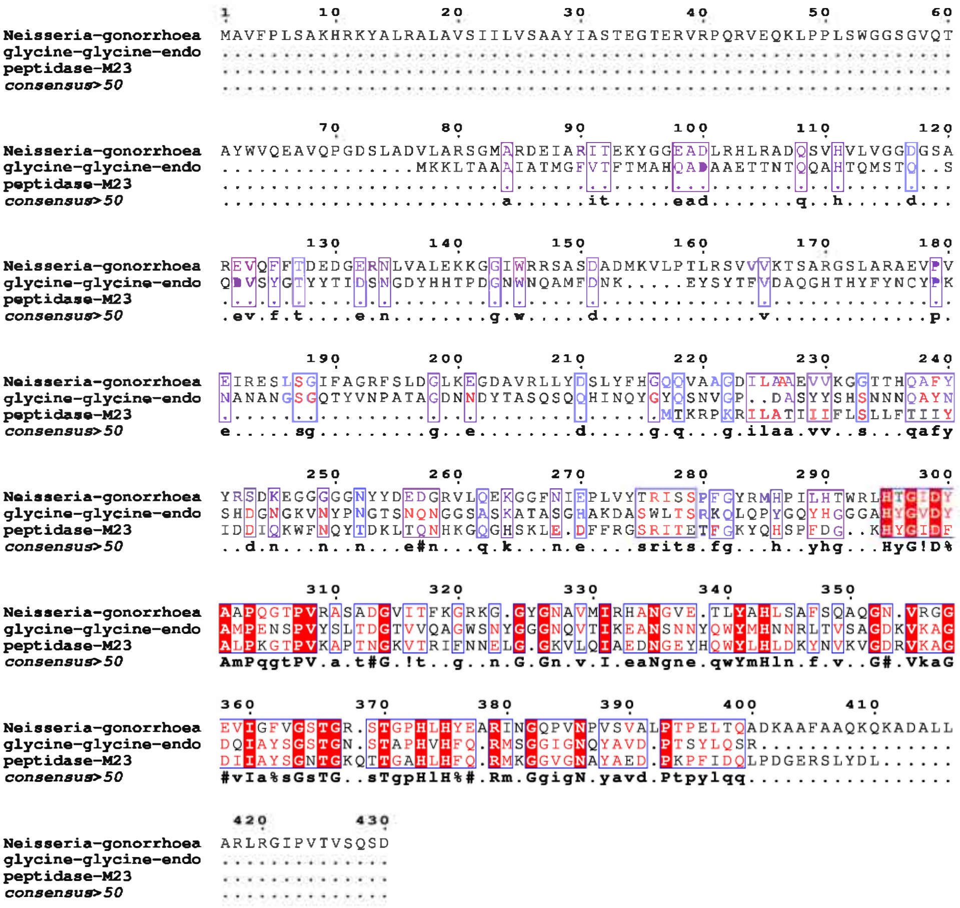
Neisseria-gonorrhoea MAVFPLSAKHRKYALRALAVSIILVSAAYIASTEGTERVRPQRVEQKLPPLSWGGSGVQT
Neisseria-gonorrhoea AYWVQEAVQPGDSLADVLARSGMARDEIARITEKYGGEADLRHLRADQSVHVLVGGDGSA
glycine-glycine-endo ...............MKKLTAAAIATMGFVTFTMAHQADAAETTNTQQAHTQMSTQ..S
consensus>50 ....a......it.....ead.......q..h.....d...
Neisseria-gonorrhoea REVQFFTDEDGERNLVALEKKGGIWRRSASDADMKVLPTLRSVVVKTSARGSLARAEVPV
glycine-glycine-endo QDVSYGTYYTIDSNGDYHHTPDGNWNQAMFDNK.....EYSYTFVDAQGHTHYFYNCYPK
consensus>50 .ev.f.t....e.n........g.w.....d.............v.............p.
Neisseria-gonorrhoea EIRESLSGIFAGRFSLDGLKEGDAVRLLYDSLYFHGQQVAAGDILAAEVVKGGTTHQAFY
glycine-glycine-endo NANANGSGQTYVNPATAGDNNDYTASQSQQHINQYGYQSNVGP..DASYYSHSNNNQAYN
peptidase-M23 ....................................MTKRPKRILATIIIFLSLLFTIIY
consensus>50 e.....sg.........g..e........d.....g.q...g.ilaa.vv..s...qafy
Neisseria-gonorrhoea YRSDKEGGGGGNYYDEDGRVLQEKGGFNIEPLVYTRISSPFGYRMHPILHTWRLHTGIDY
glycine-glycine-endo SHDGNGKVNYPNGTSNQNGGSASKATASGHAKDASWLTSRKQLQPYGQYHGGGAHYGVDY
peptidase-M23 IDDIQKWFNQYTDKLTQNHKGQGHSKLE.DFFRGSRITETFGKYQHSPFDG..KHYGIDF
consensus>50 ..d.n...n..n...e#n...q.k....n.e....srits.fg...h..yhg...HyG!D%
Neisseria-gonorrhoea AAPQGTPVRASADGVITFKGRKG.GYGNAVMIRHANGVE.TLYAHLSAFSQAQGN.VRGG
glycine-glycine-endo AMPENSPVYSLTDGTVVQAGWSNYGGGNQVTIKEANSNNYQWYMHNNRLTVSAGDKVKAG
peptidase-M23 ALPKGTPVKAPTNGKVTRIFNNELG.GKVLQIAEDNGEYHQWYLHLDKYNVKVGDRVKAG
consensus>50 AmPqgtPV.a.t#G.!t..g..n.G.Gn.v.I.eaNgne.qwYmHln.f.v..G#.VkaG
Neisseria-gonorrhoea EVIGFVGSTGR.STGPHLHYEARINGQPVNPVSVALPTPELTQADKAAFAAQKQKADALL
glycine-glycine-endo DQIAYSGSTGN.STAPHVHFQ.RMSGGIGNQYAVD.PTSYLQSR................
peptidase-M23 DIIAYSGNTGKQTTGAHLHFQ.RMKGGVGNAYAED.PKPFIDQLPDGERSLYDL......
consensus>50 #vIa%sGsTG..sTgpHlH%#.Rm.GgigN.yavd.Ptpylqq................
Neisseria-gonorrhoea ARLRGIPVTVSQSD

Fig. S6

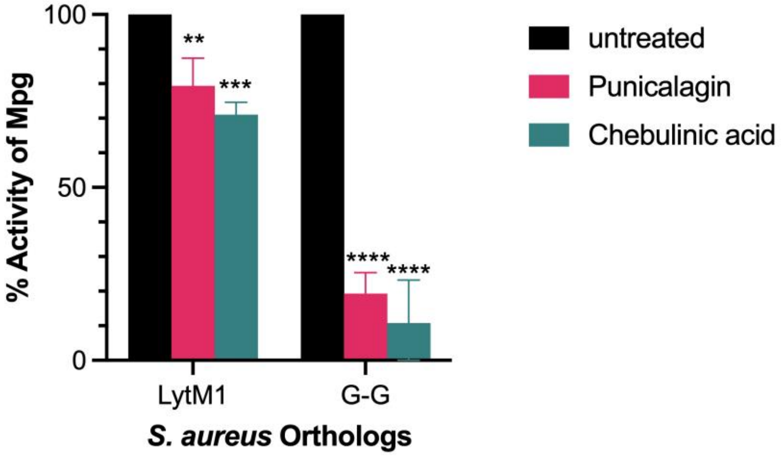

100
50
0
% Activity of Mpg
**
***
****
****
LytM1
G-G
S. aureus Orthologs
untreated
Punicalagin
Chebulinic acid

Fig. S7

A.

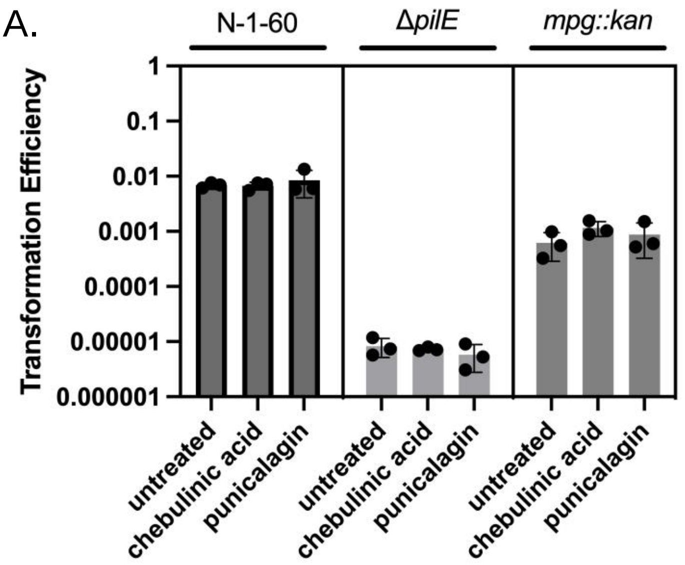
N-1-60
ΔpilE
mpg::kan
Transformation Efficiency
1
0.1
0.01
0.001
0.0001
0.00001
0.000001
untreated
chebulinic acid
punicalagin
untreated
chebulinic acid
punicalagin
untreated
chebulinic acid
punicalagin

B.

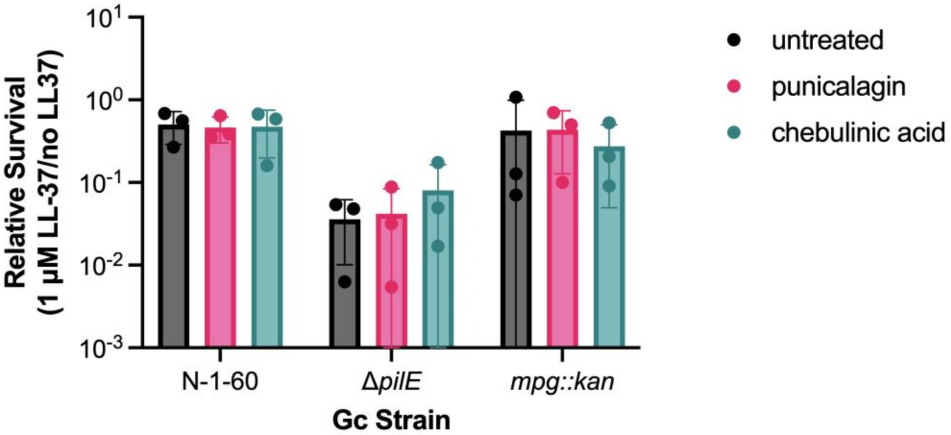
Relative Survival
(1 µM LL-37/no LL37)
10^1
10^0
10^-1
10^-2
10^-3
N-1-60
ΔpilE
mpg::kan
Gc Strain
untreated
punicalagin
chebulinic acid

Fig. S8

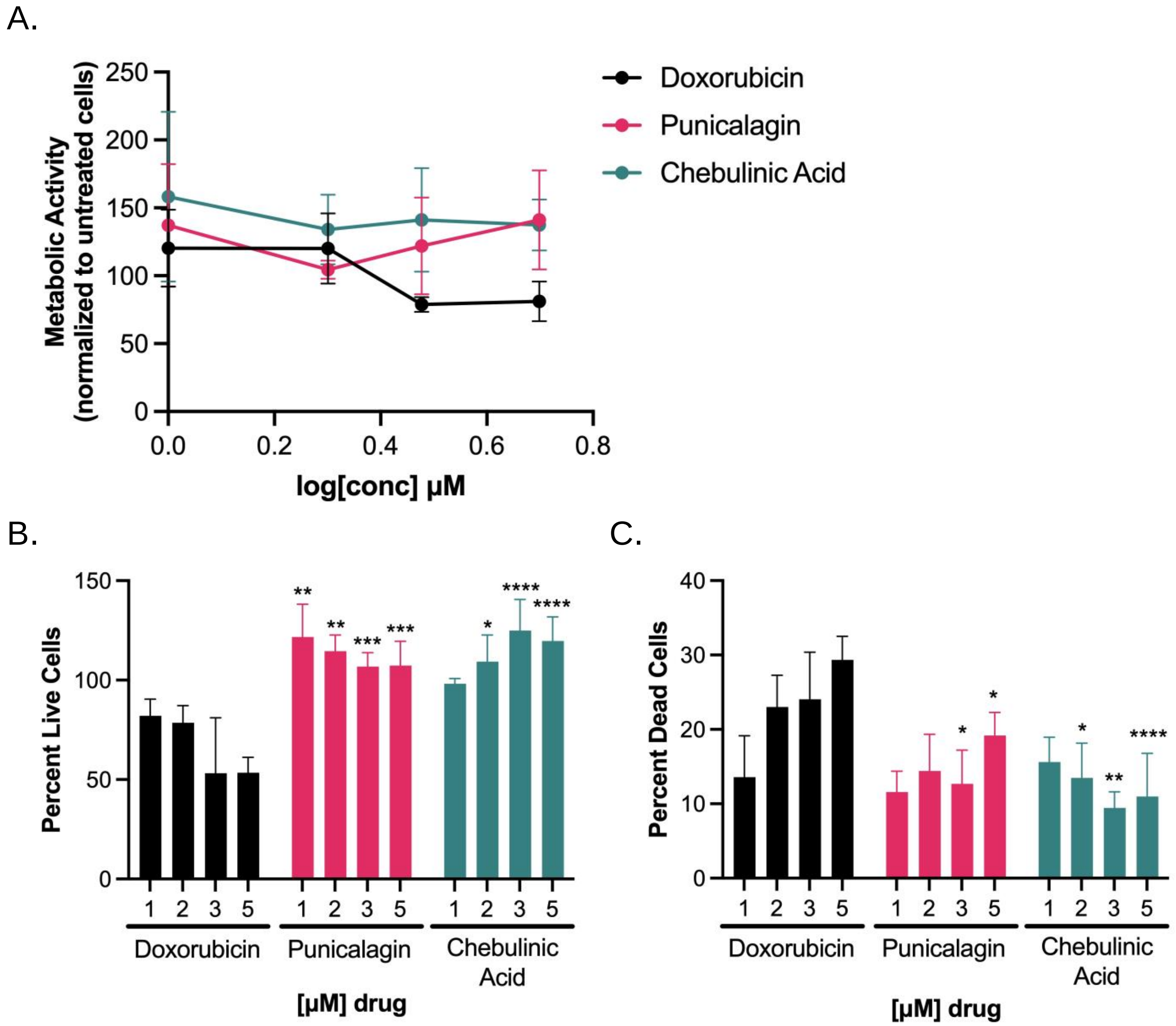
A.
Metabolic Activity (normalized to untreated cells)
250
200
150
100
50
0
0.0
0.2
0.4
0.6
0.8
log[conc] μM
Doxorubicin
Punicalagin
Chebulinic Acid
B.
Percent Live Cells
150
100
50
0
**
**
***
***
*
****
****
1 2 3 5
1 2 3 5
1 2 3 5
Doxorubicin
Punicalagin
Chebulinic Acid
[μM] drug
C.
Percent Dead Cells
40
30
20
10
0
*
*
*
**
****
1 2 3 5
1 2 3 5
1 2 3 5
Doxorubicin
Punicalagin
Chebulinic Acid
[μM] drug

Fig. S9

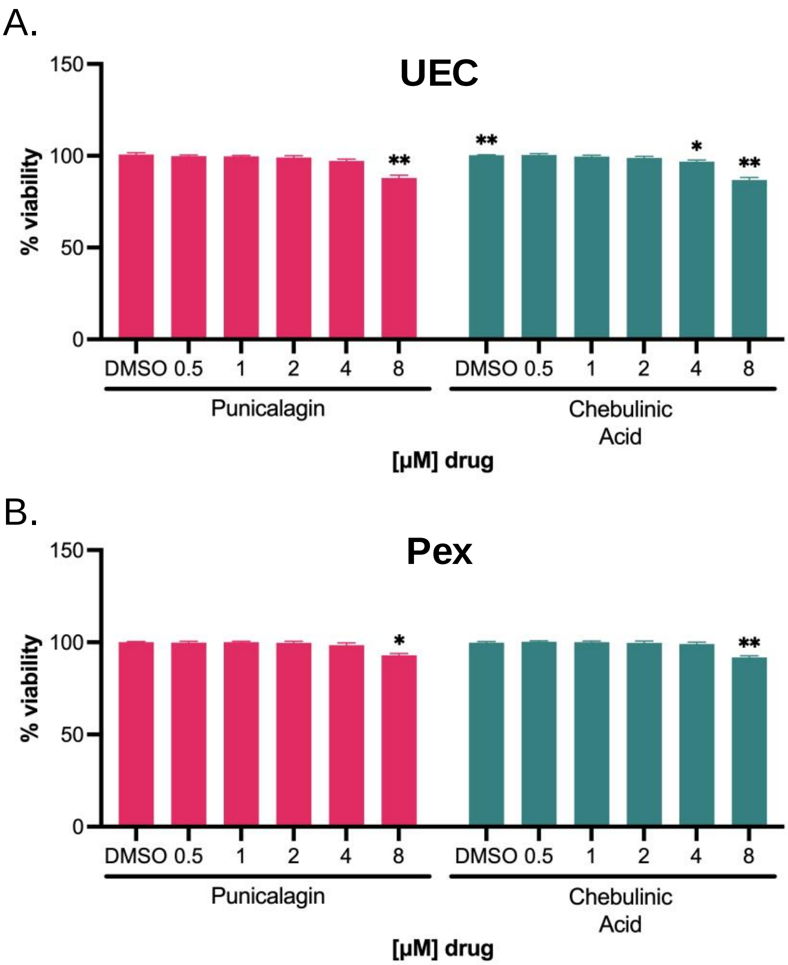
A.
UEC
150
100
50
0
% viability
**
**
*
**
DMSO 0.5 1 2 4 8
DMSO 0.5 1 2 4 8
Punicalagin
Chebulinic Acid
[μM] drug
B.
Pex
150
100
50
0
% viability
*
**
DMSO 0.5 1 2 4 8
DMSO 0.5 1 2 4 8
Punicalagin
Chebulinic Acid
[μM] drug

Supplemental Figure Legends

Supplemental Figure 1. Natural compounds related to punicalagin have activity against Mpg. A. several natural compounds. Each reaction contained 1 mM Mpg and 20 mM of indicated compound except Mpg alone. The Mpg activity is shown as related to Mpg alone activity. Data were analyzed by one-way ANOVA ($p<0.0001$) with multiple comparisons to untreated Mpg (theaflavin *, p= 0.0477; suramin *, p=0.0442; **, p=0.0018; ***, p=0.0003; ****, p<0.0001). **B.** Chemical structures of compounds with activity against Mpg.

Supplemental Figure 2. Products of punicalagin catabolism retain activity against Mpg. A. Chemical structures of punicalagin derivatives showing the reaction steps. **B.** Punicalagin analog activity against Mpg. Each reaction contained 1 µM Mpg and 20 µM of each compound except Mpg alone. Data were analyzed by one-way ANOVA ($p<0.0001$) with multiple comparisons to untreated Mpg (****, p<0.0001).

Supplemental Figure 3. Modeling of punicalagin and chebulinic acid binding to Mpg. A. Molecular docking analysis of Mpg and punicalagin or chebulinic acid by Autodock identified three binding sites, where only site 1 is predicted by Alphafold docking model. **B.** Space-filling model of Mpg-punicalagin and Mpg chebulinic acid binding poses. Molecular graphs are generated in Pymol with color scheme: Mpg in green-cyan and Zinc in yellow, punicalagin and chebulinic acid: Carbon in blue, Oxygen in red, and Hydrogen in white.

Supplemental Figure 4. Gram-negative orthologs of Mpg. Multiple sequence alignment of Mpg orthologs created using MultAlin and visualized in ESPript3.

Supplemental Figure 5. Gram-positive *S. aureus* orthologs of Mpg. Multiple sequence alignment of Mpg orthologs, created in MultAlin and visualized in ESPript3.

Supplemental Figure 6. Punicalagin and chebulinic acid show activity against Gram+ Mpg orthologs. Mpg orthologs from Gram-positive species S. aureus. LytM1, M23 peptidase family; G-G, glycine-glycine endopeptidase. In each reaction, 10 µM compound was added to 1 µM enzyme using 10 mg/ml RBB labeled Staphylococcus PG as substrate. Statistical analysis was performed using a two-Way ANOVA with multiple comparisons. Significance is shown relative to the untreated ortholog. LyM1: ***, p=0.0001; **, p=0.0029. G-G: ****, p<0.0001.

Supplemental Figure 7. Treatment with punicalagin or chebulinic acid does not alter *N. gonorrhoeae* transformation efficiency or resistance to LL-37 killing. A. Transformation efficiency assays were performed using 50ng of pSY6 plasmid DNA, which confers resistance to nalidixic acid. Cells were treated with DNA for 20 minutes followed by a 10-minute DNase I treatment. Efficiency was calculated by comparing the number of nalidixic acid-resistant cells to the total number of cells plated. **B.** Relative survival of *N. gonorrhoeae* exposed to LL-37 in the presence or absence of punicalagin or chebulinic acid.

Supplemental Figure 8. Punicalagin and chebulinic acid do not decrease metabolic activity or lead to cytotoxicity in HeLa cells. A. MTS assay measuring metabolic activity of HeLa cells grown in the presence of increasing concentrations (1 µM-5 µM) anti-Mpg compounds. Doxorubicin serves as a positive control for decreased cellular metabolism. **B.** Live/Dead cytotoxicity assay. HeLa cells were grown with increasing concentrations of anti-Mpg compounds. Live cells were quantified using calcein-AM (1 µM) staining. Calcein-AM was measured at an excitation of 485nm and emission of 530nm. Percent live cells was calculated relative to untreated cells stained with Calcein-AM. Statistical analysis was performed using a two-way ANOVA with multiple comparisons to doxorubicin at indicated concentrations. Punicalagin: 1 µM, **, $p=0.0033$; 2 µM, **, $p=0.0074$; 3 µM and 5 µM, ***, $p=0.0001$. Chebulinic acid: 2 µM, *, $p=0.0227$; 3 µM and 5 µM, ****, $p<0.0001$. **C.** Live/Dead cytotoxicity assay. HeLa cells were grown with increasing concentrations of anti-Mpg compounds. Live cells were quantified using Ethidium homodimer (EthD-1; 2 µM) staining. EthD-1 was measured at an excitation of 485nm and emission of 530nm. Percent dead cells was calculated relative to 0.01% saponin-treated cells stained with EthD-1. Statistical analysis was performed using a two-way ANOVA with multiple comparisons to doxorubicin at indicated concentrations. Punicalagin: 3 µM, *, $p=0.0115$; 5 µM, *, $p=0.0248$. Chebulinic acid: 2 µM, *, $p=0.0359$; 3 µM, **, $p=0.0013$; 5 µM, ****, $p<0.0001$.

Supplemental Figure 9. Punicalagin and chebulinic acid do not decrease viability of UEC or Pex cells. A. UEC or **B.** Pex cells incubated for 24h with noted agent before rinsing and addition of fluorescence assay reagent using the Abcam Viability Assay Kit. Cells were incubated for 1h at 37ºC before reading 530/25 excitation; 645/40 emission. Increased fluorescence corresponds to increased cell viability. A paired student's t-test was used to determine statistical significance. **, $p<0.01$; *, $p<0.05$.
