# Supplementary material for: Natural compounds target the M23B zinc metallopeptidase Mpg to modulate Neisseria gonorrhoeae Type IV pilus expression
Source: mBio. 2025 Feb 25;16(4):e04027-24. doi: 10.1128/mbio.04027-24 (PMC11980366; doi:10.1128/mbio.04027-24)
Supplement: Supplemental Tables — Tables S1 to S4. [file mbio.04027-24-s0002.docx]

**Table S1. Putative punicalagin or chebulinic acid binding sites.**

| **Site** | **Amino Acid Residues** |
| --- | --- |
| 1 | H295, D299, H343, H373, H375 |
| 2 | D128, E129, D130, E132, R133, L294, T296 |
| 3 | Q108, S109, R175, E177, V178, V180, S211, Q217, Q218, V219 |
| Predicted NAG-NAM binding site | N134, V136, L138, R147, H286, L289, T291, R293 |

**Table S2. Strains and plasmids used in this study.**

| **Strain Name** | **Strain Details** | **Reference** |
| --- | --- | --- |
| N-1-60 | *N. gonorrhoeae* strain FA1090 with multisite G4 mutant 1-81-S2 pilE variant, *pilC1_PL_* | Hu LI and Yin S, et al. mBio11:10.1128/mbio.02528-20. https://doi.org/10.1128/mbio.02528-20 |
| N-1-60 *mpg::erm* | N-1-60 insertional mutant containing an erythromycin resistance cassette in place of the *mpg* (*NGO_1686*) gene | This study. |
| N-1-60 Δ*pilE* | Unmarked *pilE* deletion spanning the 6^th^ amino acid to stop codon. | Hu LI and Yin S, et al. mBio11:10.1128/mbio.02528-20. https://doi.org/10.1128/mbio.02528-20 |
| BL21 (DE3) Star | T7-based protein expression | Invitrogen |
| HL-1-22 | *N. gonorrhoeae* strain MS11 with multisite G4 mutant 1-81-S2 pilE variant, *pilC1_PL_* | This study. |

**Table S3. Strains and plasmids used in this study.**

| **Plasmid Name** | **Plasmid Details** | **Reference** |
| --- | --- | --- |
| pTwist:*mpg::erm* | pTwistAmpMC plasmid carrying a synthetic *mpg::erm* construct. Amp^r^, Erm^r^ | Twist Bioscience |
| pET28a | Expression vector with T7 promoter and N-terminal 6x-His tag and N-terminal thrombin cleavage site. Kan^r^. | Novagen |
| pET28a:*mpg* | Mpg (*N. gonorrhoeae*) with an N-terminal 6x-His tag. Kan^r^ | This study. |
| pET28a:*mpg*_Pa_ | Mpg (*P. aeruginosa* PAO1) with an N-terminal 6x-His tag. Kan^r^ | This study. |
| pET28a:*mpg_Nm_* | Mpg (*N. meningitidis* MC58) with an N-terminal 6x-His tag. Kan^r^ | This study. |
| pET28a:*mpg*_Ec_ | Mpg (*E. coli* K12 MG1655) with an N-terminal 6x-His tag. Kan^r^ | This study. |
| pET28a:*mpg_Ab_* | Mpg (*A. baumannii* ATCC17978) with an N-terminal 6x-His tag. Kan^r^ | This study. |
| pET28a:*mpg_Vc_* | Mpg (*V. cholerae* O395) with an N-terminal 6x-His tag. Kan^r^ | This study. |
| pET28a:*mpg_Sa_* | *S. aureus* M23 family metallopeptidase with an N-terminal 6x-His tag. Kan^r^ | This study. |
| pET28a:*lytM_Sa_* | *S. aureus* glycine-glycine endopeptidase LytM with an N-terminal 6x-His tag. Kan^r^ | This study. |
| pSY6 | Plasmid containing *N.* gonorrhoeae-derived 10-kb PstI fragment which confers resistance to nalidixic acid through mutation of the *gyrB* gene. | Stein DC, Danaher RJ, Cook TM. Antimicrob Agents Chemother https://doi.org/10.1128/aac.35.4.622 |

**Table S4. Primers used in this study.**

| **Mpg orthologs** | **Protein ID** | **Species** | **Primers** |
| --- | --- | --- | --- |
| Peptidoglycan DD-metalloendopeptidase | WP_003085274.1 | *P. aeruginosa* PAO1 | F: aaagctagcaagaggaccacgctcaatctg  R: aaaaagcttgtagcgcggcacgatcagcg |
| M23 family metallopeptidase | WP_002224862.1 | *N. meningitidis* MC58 | F: aaagctagcgagaggacggagcgcgtcag  R: aaaaagcttcaaacttcaatccgattgcgac |
| Murein DD-endopeptidase MepM | NP_416370.2 | *E. coli* K12 MG1655 | F:aaagctagcgtttatcaccgtgatgccacgcc  R: aaaaagcttctgactgcgtaccggctgcg |
| M23 family metallopeptidase | AKQ25332.1 | *A. baumannii* ATCC17978 | F: aaagctagcgattatcaaaatattaaccaatcc  R: aaaaagcttccttgaccttggaggcaatg |
| Peptidoglycan DD-metalloendopeptidase ShyA | WP_001882946.1 | *V. cholerae* O395 | F: aaagctagcctaaacagtcccacgcggcaac  R: aaaaagcttcggggctttgtattcacactcg |
| M23 family metallopeptidase | OOC91870.1 | *S. aureus*  ATCC25923 | F:aaagctagcgatgacatacaaaaatggtttaac  R: aaaaagcttctacaaatcatataggctacgttc |
| Glycine-glycine endopeptidase LytM | WP_086896458.1 | *S. aureus* RN450 | F:aaagctagcgcagaaacgacaaacacccaac  R: aaaaagcttgggatttactgtattatctac |
